# Ruminococcin C, an anti-clostridial sactipeptide produced by a prominent member of the human microbiota *Ruminococcus gnavus*

**DOI:** 10.1074/jbc.RA119.009416

**Published:** 2019-07-23

**Authors:** Clémence Balty, Alain Guillot, Laura Fradale, Clémence Brewee, Mylène Boulay, Xavier Kubiak, Alhosna Benjdia, Olivier Berteau

**Affiliations:** Micalis Institute, ChemSyBio, INRA, AgroParisTech, Université Paris-Saclay, 78350 Jouy-en-Josas, France

**Keywords:** antimicrobial peptide (AMP), enzyme, peptide biosynthesis, natural product biosynthesis, radical, antibiotics, microbiome, radical AdoMet enzyme, radical SAM enzyme, RiPP, ruminococcin, ruminococcin C

## Abstract

The human microbiota plays a central role in human physiology. This complex ecosystem is a promising but untapped source of bioactive compounds and antibiotics that are critical for its homeostasis. However, we still have a very limited knowledge of its metabolic and biosynthetic capabilities. Here we investigated an enigmatic biosynthetic gene cluster identified previously in the human gut symbiont *Ruminococcus gnavus*. This gene cluster which encodes notably for peptide precursors and putative radical SAM enzymes, has been proposed to be responsible for the biosynthesis of ruminococcin *C* (RumC), a ribosomally synthesized and posttranslationally modified peptide (RiPP) with potent activity against the human pathogen *Clostridium perfringens*. By combining *in vivo* and *in vitro* approaches, including recombinant expression and purification of the respective peptides and proteins, enzymatic assays, and LC-MS analyses, we determined that RumC is a sulfur-to–α-carbon thioether-containing peptide (sactipeptide) with an unusual architecture. Moreover, our results support that formation of the thioether bridges follows a processive order, providing mechanistic insights into how radical SAM (AdoMet) enzymes install posttranslational modifications in RiPPs. We also found that the presence of thioether bridges and removal of the leader peptide are required for RumC's antimicrobial activity. In summary, our findings provide evidence that production of the anti-*Clostridium* peptide RumC depends on an *R. gnavus* operon encoding five potential RumC precursor peptides and two radical SAM enzymes, uncover key RumC structural features, and delineate the sequence of posttranslational modifications leading to its formation and antimicrobial activity.

## Introduction

Despite its growing importance in biology, study of the human microbiome remains a challenging area of investigation. Recently, it has emerged that environmental rather than genetic factors play a major role in shaping this complex ecosystem, one of the densest on earth (1). Among the molecular determinants underpinning the normal equilibrium within the microbiota (eubiosis), it has been predicted that antimicrobial substances should play a central role (2, 3). However, to date, only few antimicrobial peptides from the human microbiome have been identified and characterized. Among these, ribosomally synthesized and posttranslationally modified peptides (RiPPs)^3^ represent a growing family of natural products that has attracted considerable interest (4), propelled by the need for novel antibiotics and their involvement in homeostasis of the microbiota (5, 6). *Ruminococcus gnavus* is an inhabitant of the human digestive tract, with some strains able to degrade mucins and host glycans (7), similar to the prominent gut symbiont *Bacteroides thetaiotaomicron* (8, 9). However, despite its widespread distribution in humans and its potential role in human physiology, the metabolic properties of this Gram-positive bacterium are just starting to be unraveled. For instance, *R. gnavus* has been shown to produce several antimicrobial peptides, including RumA (10) and RumC (11, 12), which are both active against *Clostridium* species. RumA has been shown to be a RiPP containing three lanthionine bridges (*i.e.* three β-thioether bonds) and, thus, to belong to the large class of lanthipeptides (13). Lanthipeptides are well-known to exhibit a wide range of biological activities spanning from antimicrobial activities to antiviral, antinociceptive, and antiallodynic functions (14). Some lanthipeptides, such as nisin, exert their antimicrobial activity by binding to lipid II (15). However, for the vast majority of them, we still have limited knowledge of their mode of action. Lanthionine bridges are installed by a two-step mechanism involving dehydration of a Ser or Thr residue followed by stereoselective intramolecular Michael addition of the thiol group of a remote Cys residue. Intriguingly, other thioether-containing peptides called sactipeptides (sulfur-to–α-carbon thioether-containing peptides) have been described recently (5, 16). In contrast to lanthipeptides, formation of thioether bridges in sactipeptides involves a radical-based mechanism catalyzed by radical SAM enzymes (5, 6, 17) and leads to the formation of α-thioether bridges (18). By combining *in vivo* and *in vitro* approaches, we succeeded to unveil the structure of the elusive bacteriocin RumC. Our data show that RumC is a sactipeptide, the first one isolated from the human microbiota, and that it possesses a distinctive architecture. In addition, our study sheds new light on how radical SAM enzymes install posttranslational modifications in RiPPs.

## Results

### In vivo production of C1 & C2 peptides

**Figure 1.**
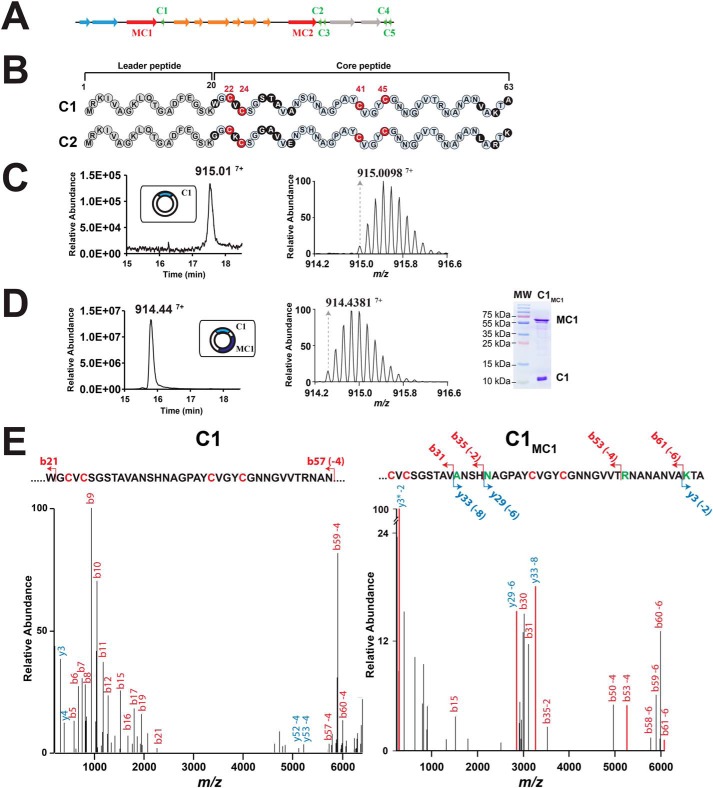
**Expression of the C1 peptide alone or with the radical SAM enzyme MC1 (C1_MC1_) in *E. coli*.** *A*, the gene cluster involved in RumC biosynthesis. *Green*, *c1* to *c5*, genes predicted to encode RumC peptides; *red*, *mc1* and *mc2*, genes predicted to encode tailoring radical SAM enzymes; *orange*, genes predicted to be involved in immunity; *blue* and *gray*, putative exporters. *B*, sequences of the C1 and C2 peptides. Amino acid residues from the predicted leader sequence are in *gray*. Conserved amino acid residues from the core sequence are in light *blue*, and nonconserved amino acid residues are in *black*. The four conserved cysteine residues are in *red. Numbers* indicate relative position to the sequence. *C*, LC-MS analysis of the C1 peptide. MS profile analysis (*left panel*) and isotopic distribution (*right panel*) of the C1 peptide. *D*, analysis of C1_MC1_ peptide. MS profile analysis (*left panel*), isotopic distribution (*center panel*), and SDS-PAGE analysis (*right panel*) of the C1_MC1_ peptide. *MW*, molecular weight. *E*, LC-MS/MS analysis of the C1 (*left panel*) and C1_MC1_ (*right panel*) peptides. Diagnostic *y* and *b* ion fragments are indicated on the primary sequence with the associated loss of hydrogen atoms (mass shift of Δ_m −_2, −4, −6, and −8 Da). Fragments are represented by an *arrow*. Cysteine and modified amino acids residues are indicated in *red* and *green*, respectively. The *asterisk* indicates the loss of ammonia.

The RumC biosynthetic cluster contains a complex set of genes suggestive of gene duplication events and rearrangements (11) (Fig. 1*A*). Notably, it includes five small genes (*c1* to *c5*, <200 bp) encoding putative precursors of RumC (hereafter referred to as C1 to C5). These peptides are 63 amino acids long (identity from 70% to 87%) and characterized by the presence of four strictly conserved cysteine residues (Fig. 1*B* and Fig. S1) and a highly conserved C-terminal region (residues 30–60). Besides putative proteases and ABC transporters, only two tailoring enzymes, RumMC1 and RumMC2 (hereafter referred to as MC1 and MC2), are predicated in the RumC biosynthetic operon (Fig. 1*A*). These enzymes share extensive sequence identity (>95%) and possess several conserved cysteine motifs: C*X*3C*X*2C and C*X*13G*X*4C*X*36C*X*2C*X*5C*X*2C*X*18C (Fig. S2), characteristic of members of the large class of SPASM-domain radical SAM enzymes (5, 19–24).

Several recent reports have shown that RiPPs produced by Gram-positive bacteria can be efficiently modified by their cognate radical SAM enzymes when expressed in *Escherichia coli* (25–27). For heterologous expression of RumC, we selected *c1* and *c2*, as these two genes have been shown to be highly induced in *R. gnavus* when this bacterium colonizes the digestive tract of rats (11, 12). In addition, both genes are cotranscribed with the *mc1* and *mc2* genes, coding for the respective radical SAM enzymes MC1 and MC2 (Fig. 1*A*). We expressed C1 and C2 as His tag fusion peptides with a tobacco etch virus cleavage site to perform their expression in *E. coli* and purification by affinity chromatography.

**Figure 2.**
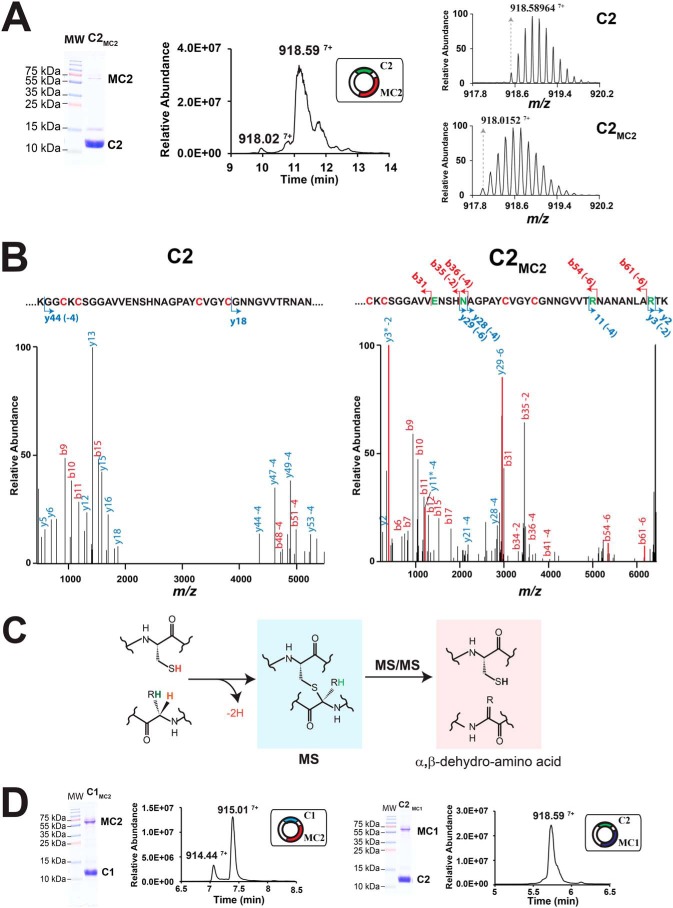
**Analysis of the C2 peptide expressed with the radical SAM enzyme MC2 (C2_MC2_) in *E. coli*.** *A*, SDS-PAGE analysis (*left panel*), MS profile analysis (*center panel*), and isotopic distribution (*right panel*) of the C2 and C2_MC2_ peptides. *MW*, molecular weight. *B*, LC-MS/MS analysis of the C2 (*left panel*) and C2_MC2_ (*right panel*) peptides. Diagnostic *y* and *b* ion fragments are indicated on the primary sequence with the associated loss of hydrogen atoms (mass shift of Δ_m −_2, −4, −6, and −8 Da). Fragments are represented by an *arrow*. Cysteine and modified amino acids residues are indicated in *red* and *green*, respectively. The full sequence is shown in Figure 1. *C*, proposed mechanism for Cα-thioether fragmentation during LC-MS/MS analysis. As shown, during LC-MS/MS analysis, thioether bridges are opened, leading to formation of a cysteine and an α,β-dehydro-amino acid residue. *D*, analysis of the C1_MC2_ and C2_MC1_ peptides expressed in *E. coli. Left panel*, SDS-PAGE and MS profile analysis of C1_MC2_. *Right panel*, SDS-PAGE and MS profile analysis of C2_MC1_.

As shown, the C1 peptide had a mass of [M+7H]^7+^ 915.01, compared with its theoretical mass of [M+7H]^7+^ 915.58 (Fig. 1*C* and Table S1). This mass shift of −0.57 Da corresponds, when taking into account the charge of the ion (7+), to a Δ_m_ of −4.03 Da, supporting the loss of four H atoms. In addition, the characteristic ions *b21*, *b57* (−4 Da), *b58* (−4 Da), and *b59* (−4 Da), obtained during LC-MS/MS analysis (Fig. 1*E* and Table S2), were consistent with the formation of two disulfide bridges between the four cysteine residues present within the peptide sequence. Similarly, after purification, the C2 peptide was mainly under an oxidized form ([M+7H]^7+^_obs_ 918.59), although low amounts of the linear form ([M+7H]^7+^_obs_ 919.16) were in evidence (Fig. 2*A* and Fig. S3 and Table S2). The fragmentation pattern of the C2 peptide was essentially identical to the one of the C1 peptide (Fig. 2*B* and Fig. S3). In addition, the fragments *y44* (−4 Da) and *y18* allowed us to precisely pinpoint the mass loss on the region containing the four conserved cysteine residues. Finally, no *b* or *y* ions from the cysteine-rich domain were present in the MS spectrum, confirming the formation of internal disulfide bridges. Thus, when expressed in *E. coli*, both peptides were essentially purified under an oxidized form with two disulfide bridges.

### Four thioether bonds in RumC1 and RumC2

When we coexpressed the C1 and C2 peptides with their cognate radical SAM enzymes (MC1 and MC2, respectively), we obtained two novel peptides, hereafter referred to as C1_MC1_ and C2_MC2_. After purification by Ni-NTA chromatography and treatment with tobacco etch virus protease, both C1_MC1_ and C2_MC2_ were essentially pure and copurified with the radical SAM enzyme MC1 or MC2 (Figs. 1*D* and 2*A*). The C1_MC1_ peptide had a mass of [M+7H]^7+^_obs_ 914.44 (Fig. 1*D* and Table S1), indicating a mass loss of Δ_m_ = −8.06 Da compared with the expected mass of the C1 peptide. In addition, LC-MS/MS analysis of the C1_MC1_ peptide revealed a distinct fragmentation pattern compared with the C1 peptide, with many *b* and *y* ions scattered in the C1_MC1_ spectrum (Fig. 1*E*). Mass shifts of Δ_m_ = −2.01 Da, −4.02 Da, −6.04 Da, and −8.06 Da were measured on the fragments *y3*, *b53*, *y29*, and *y33*, respectively (Fig. 1*E* and Table S2), pointing to Ala^31^, Asn^35^, Arg^53^, and Lys^61^ as modified residues. Despite the low amounts obtained, LC-MS/MS analysis of C2_MC2_ (Fig. 2*B*) exhibited the same characteristic fragments than those observed for C1_MC1_. The additional *b* and *y* ions *b31*, *b35* (−2 Da), *y29* (−6 Da), *y28* (−4 Da), *y11* (−4 Da), *y3* (−2 Da), and *y2* allowed us to accurately pinpoint Glu^31^, Asn^35^, Arg^53^, and Arg^61^ as modified residues in the C2_MC2_ peptide, (Fig. 2*B* and Table S2).

C_α_-thioether bridges are well-known to undergo facile retroelimination and tautomerization during LC-MS/MS analysis, leading to the formation of α,β-dehydro-amino acids, characterized by a mass loss of Δ_m_ = −2.01 Da (Fig. 2*C*). In addition, the new amide bonds formed are extremely labile at low voltage, leading to easy breakage and mapping by LC-MS/MS (26, 28, 29). Collectively, our data thus establish that C1_MC1_ and C2_MC2_ contain four α-thioether bridges involving Ala^31^, Asn^35^, Arg^53^, and Lys^61^ in C1_MC1_ or Glu^31^, Asn^35^, Arg^53^, and Arg^61^ in C2_MC2_.

The fact that only a low amount of the C2 peptide was modified when coexpressed with MC2 was intriguing (Fig. 2*A*), considering that C1 was fully matured when coexpressed with MC1 (Fig. 1*D*) and the high sequence identity between the two enzymes and peptides. To distinguish between a peptide and an enzyme issue, we coexpressed C1 with MC2 or C2 with MC1 in *E. coli* (Fig. 2*D*). As shown, MC1 was not able to modify C2 *in vivo*, whereas MC2 was active toward C1, albeit with a lower efficiency than MC1 (Fig. 2*D*). Interestingly, the C1_MC2_ peptide had the same spectroscopic signature than the C1_MC1_ peptide, supporting that both peptides contain the same posttranslational modifications. Altogether, these data support that both MC1 and MC2 install the same posttranslational modifications and that the low amount of modified C2 peptide produced (*i.e.* C2_MC2_ and C2_MC1_), is mainly due to its sequence.

### Structure of RumC2

**Figure 3.**
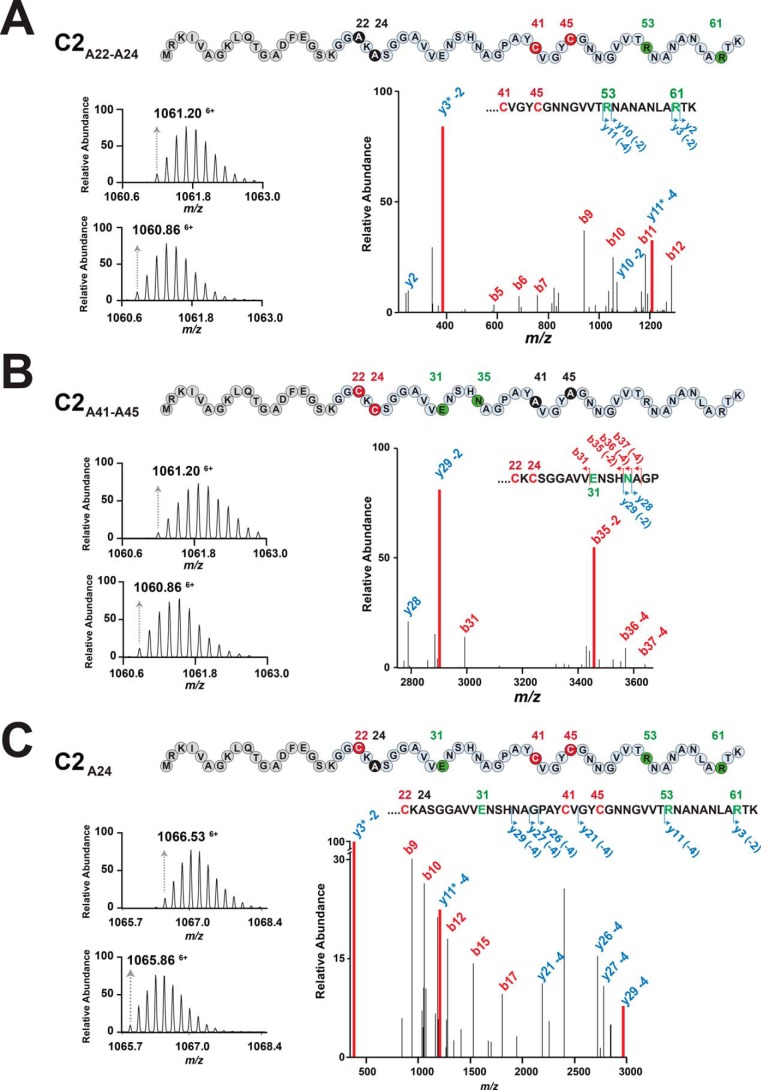
*A–C*, LC-MS/MS analysis of the C2_A22-A24_ (*A*), C2_A41-A45_ (*B*), and C2_A24_ (*C*) peptides after *in vitro* incubation with the radical SAM enzyme MC2. The MS spectrum of the oxidized substrate (*top left panel*), the product (*bottom left panel*), and the MS/MS spectrum of the product formed (*right panel*) are shown for each peptide. The corresponding peptide sequences are indicated above each panel.

To determine the connectivity between the α-positions of the four target residues (*i.e.* Ala^31^, Asn^35^, Arg^53^, and Lys^61^ in C1 or Glu^31^, Asn^35^, Arg^53^, and Arg^61^ in C2) and the four cysteine residues (*i.e.* Cys^22^, Cys^24^, Cys^41^, and Cys^45^), we devised an *in vitro* strategy. As shown above, C2 was efficiently produced without modification in *E. coli* and was thus a suitable source of substrate for *in vitro* experiments. In addition, we generated the three cysteine variants C2_A22A24_, C2_A41A45_, and C2_A24_, in which the corresponding cysteine residues Cys^22^ and Cys^24^, Cys^41^ and Cys^45^, and Cys^24^ were replaced with alanine residues, respectively (Fig. 3). As shown, after purification, the C2_A22A24_ variant had a mass of [M+6H]^6+^_obs_ 1061.20, indicating the formation of one disulfide bridge (Fig. 3*A* and Fig. S4). After incubation with MC2, the peptide mass shifted to [M+6H]^6+^_obs_ 1060.86 (Δ_m_ = −4.02 Da), consistent with the formation of two thioether bridges. LC-MS/MS analysis showed that only the residues Arg^53^ and Arg^61^ were modified (Fig. 3*A*, *right panel*, and Table S3), supporting that both residues are linked to Cys^41^ and Cys^45^.

Incubation of the C2_A41A45_ variant with MC2 led to a mass shift from [M+6H]^6+^_obs_ 1061.20 (oxidized form) to 1060.86 (Δ_m_ = −4.02 Da) (Fig. 3*B*, *left panel*) consistent with the formation of two thioether bridges. LC-MS/MS analysis located dehydrogenation on Glu^31^ and Asn^35^, supporting that both residues were linked to Cys^22^ and Cys^24^ (Table S3). Thus, in contrast to all known sactipeptides such as subtilosin A (18, 29, 30), thuricin CD (31), and thurincin H (28), RumC2 contains two hairpin-like domains: one domain with Cys^22^ and Cys^24^ connected to Glu^31^ and Asn^35^ and a second domain with Cys^41^ and Cys^45^ connected to Arg^53^ and Arg^61^.

When we assayed the C2_A24_ variant, its mass shifted from [M+6H]^6+^_obs_ 1066.53 (oxidized form) to 1065.86, consistent with the formation of three thioether bridges. LC-MS/MS analysis showed that these three thioether bridges involved Arg^53^, Arg^61^, and likely Glu^31^ but not Asn^35^ (Fig. 3*C* and Table S4), supporting that Glu^31^ was connected to Cys^22^. We failed to express a fourth variant in which Cys^22^ was replaced with an alanine residue (*i.e.* the C2_A22_ peptide). However, we succeeded to obtain small amounts of the corresponding C1 mutant (*i.e.* the C1_A22_ peptide) coexpressed with MC1. Surprisingly, with this variant, LC-MS/MS analysis was consistent with the formation of a thioether bridge between Cys^24^ and Ala^31^ (Fig. S5 and Table S4). This last result supports that, in the absence of the target cysteine residue, thioether bridges might form with a nearby cysteine residue.

**Figure 4.**
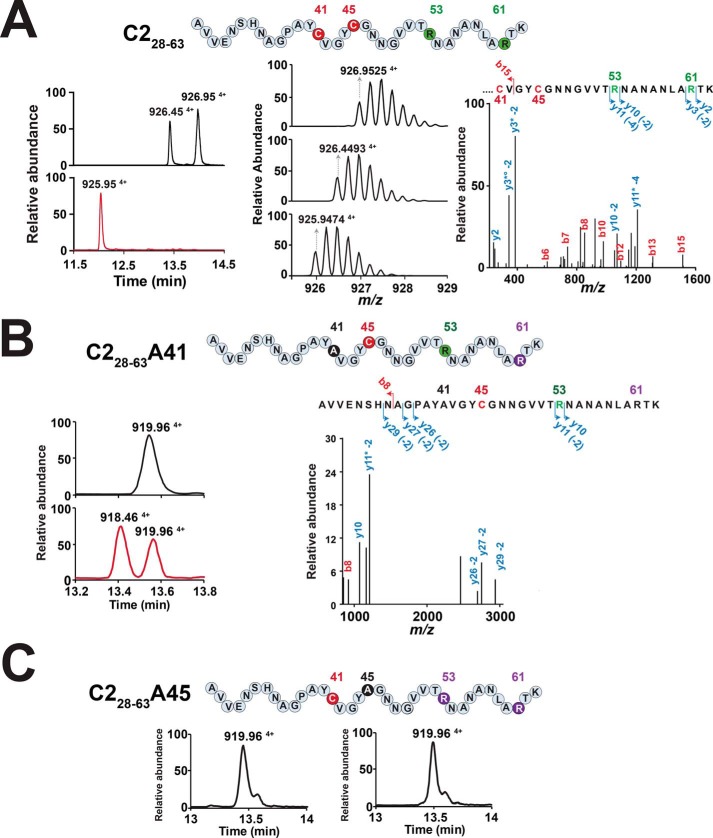
**LC-MS/MS analysis of the C2_28–63_, C2_28–63_A41, and C2_28–63_A45 peptides after *in vitro* incubation with the MC2 enzyme.** *A*, MS profile analysis of the C2_28–63_ peptide at T0 (*top left pane*l) and after 4 h (*bottom left panel*) incubation with MC2. *Center panel* from *top* to *bottom*, MS spectra of the C_28–63_ peptide, the C_28–63_ peptide (oxidized form), and the product formed. *Right panel*, MS/MS spectrum of the reaction product. Cysteine and modified amino acids residues are indicated in *red* and *green*, respectively. The full sequence is shown in Figure 1. *B*, MS profile analysis of the C2_28–63_A41 peptide at T0 (*top left pane*l) and after 4 h (*bottom left panel*) of incubation with MC2. *Right panel*, MS/MS spectrum of the reaction product. *C*, MS profile analysis of the C2_28–63_A45 peptide at T0 (*left pane*l) and after 4 h (*right panel*) of incubation with MC2. Peptide sequences are indicated above each panel. The *asterisk* indicates the loss of ammonia, and ° indicates the loss of H_2_O.

To accurately determine the connectivity of the thioether bridges, we designed, based on the C2 sequence, shorter peptide substrates containing either the first (*i.e.* residues 1–40, C2_1–40_) or the second hairpin domain (*i.e.* residues 28–63, C2_28–63_) and assayed them with MC2 *in vitro*. The C2_1–40_ peptide proved to be an extremely poor substrate and was not amenable to LC-MS/MS analysis. However, the C2_28–63_ peptide was efficiently converted by MC2, as shown by disappearance of the linear ([M+4H]^4+^_obs_ 926.95) and oxidized ([M+4H]^4+^_obs_ 926.45) forms during reaction and concomitant production of a novel peptide ([M+4H]^4+^_obs_ 925.95) (Fig. 4*A* and Table S1). LC-MS/MS analysis confirmed the formation of two thioether bridges located on Arg^53^ and Arg^61^ (numbered as in the C2 sequence), a result in agreement with the ones obtained with the full-length-peptide (Fig. 2 and Table S5). When we replaced Cys^41^ with an alanine residue, incubation of the C2_28–63_A_41_ variant with MC2 led to a product containing a single thioether bridge between Cys^45^ and Arg^53^ (Fig. 4*B*). Intriguingly, the C2_28–63_A_45_ variant did not lead to formation of any product when incubated with MC2 (Fig. 4*C*). Similarly, no product was obtained with the double mutant C2_28–63_A_41_A_45_. These results support a sequential order for formation of the thioether bridges, with the Cys^45^-Arg^53^ bridge being required first before formation of the Cys^41^-Arg^61^ bridge could take place.

### Sequential formation of the thioether bridges in RumC2

**Figure 5.**
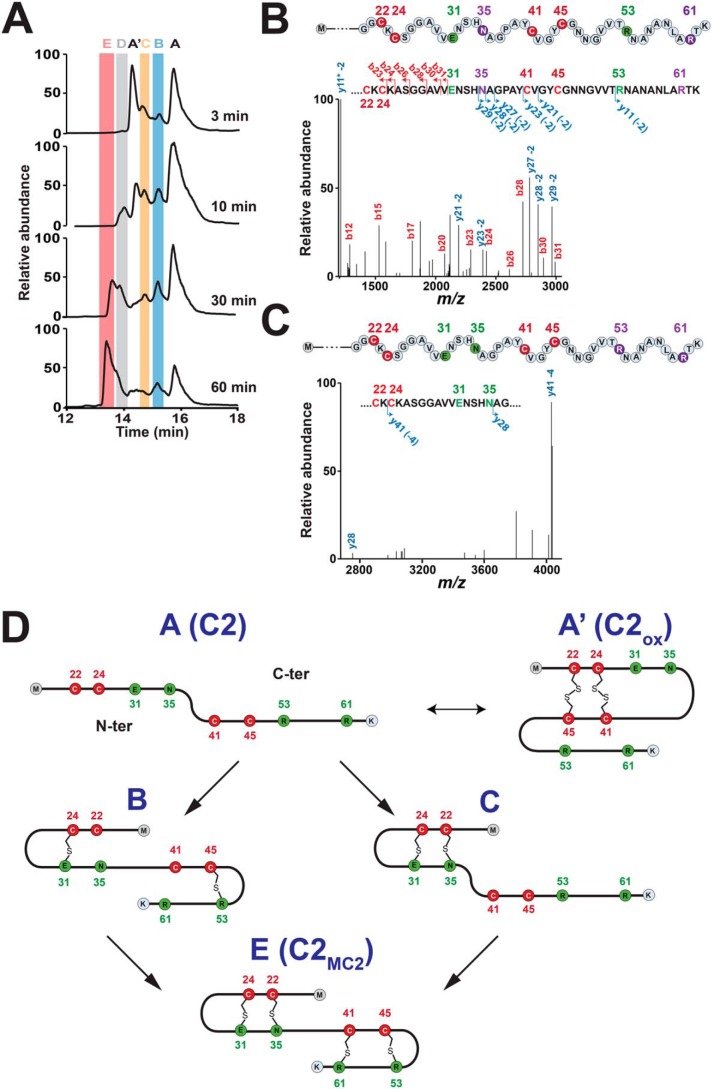
**Time course analysis of the C2 peptide incubated with the radical SAM enzyme MC2.** *A*, MS profile analysis of the C2 peptide after (*top* to *bottom*) 3-, 10-, 30-, and 60-min incubation with MC2. Each reaction intermediate is highlighted with a different color and letter. A, [M+7H]^7+^ = 919.16 (C2, linear form); A′, [M+7H]^7+^ = 918.58 (C2, oxidized form); B, [M+7H]^7+^ = 918.58; C, [M+7H]^7+^ = 918.58; D, ND; E, [M+7H]^7+^ = 918.01. *B*, LC-MS/MS analysis of intermediate B. Relevant fragments are indicated. The number of the fragment and the mass shift measured are indicated. Cysteine and modified amino acids residues are labeled in *red* and *green*, respectively. Residues not engaged in thioether bridges are in *purple*. The *asterisk* indicates the loss of ammonia. *C*, LC-MS/MS analysis of intermediate C. *D*, proposed sequential order for the formation of thioether bridges in RumC2. Starting from the precursor A, the first thioether bridges (*i.e.* the Cys^24^-Glu^31^ and Cys^45^-Arg^53^ bridges) are installed in each hairpin domain (intermediate B). Then the second thioether bridges, Cys^22^-Asn^35^ (intermediate C) and Cys^41^-Arg^61^, are formed, leading to production of the mature peptide C2_MC2_ (species E) with two hairpin domains.

To get better knowledge regarding the sequential formation of the thioether bridges and the structure of RumC2, we performed a kinetics analysis by LC-MS/MS. Using an optimized gradient and short reaction times, we were able to identify and characterize several C2 reaction intermediates (Fig. 5*A*). As shown, shortly after the reaction was initiated by addition of sodium dithionite, the substrate that existed under two forms, species A (linear form, [M+7H]^7+^_obs_ 919.16) and A′ (oxidized form, [M+7H]^7+^_obs_ 918.58), was converted into two novel species: B ([M+7H]^7+^_obs_ 918.58) and C ([M+7H]^7+^_obs_ 918.58). Species B and C were characterized as reaction intermediates containing two thioether bridges involving Glu^31^ and Arg^53^ (Fig. 5*B*) and Glu^31^ and Asn^35^ (Fig. 5*C*), respectively. After 60 min, species A and A′ had almost totally disappeared, whereas the main species, E ([M+7H]^7+^_obs_ 918.01), was identified as the mature C2_MC2_ with four thioether bridges (Fig. 5*D*). The transient species D could not be unambiguously assigned, but LC-MS/MS analysis was consistent with a reaction intermediate containing three thioether bridges *en route* to conversion into species E.

Collectively, these experiments allowed us to propose a model for formation of the thioether bridges in RumC based on the following evidence. First, thioether bridges in the N- and C-terminal domains are installed independently, as shown by the experiments performed with the C2_A22A24_ and C2_A41A45_ peptides (Fig. 3) and with the truncated C2_28–63_ peptide (Fig. 4). This conclusion is also supported by the identification of reaction intermediates having a single thioether bridge in each domain (*i.e.* intermediate B with the Cys^24^-Glu^31^ and Cys^45^-Arg^53^ bridges) or having two thioether bridges in one single domain (*i.e.* intermediate C with the Cys^24^-Glu^31^ and Cys^22^-Asn^35^ bridges in the N-terminal domain) (Fig. 5). Second, formation of the thioether bridges follows an N-to-C direction. Indeed, our data support that the Cys^45^-Arg^53^ bridge is formed before the Cys^41^-Arg^61^ bridge in the C-terminal domain (Fig. 4). Similarly, for the N-terminal domain, we identified several reaction intermediates having the Cys^24^-Glu^31^ bridge (*e.g.* intermediates B, C, and E) but none with only the Cys^22^-Asn^35^ bridge.

### Stereochemistry of the α-carbon atoms in RumC1

The vast majority of known sactipeptides, such as subtilosin A (18, 29) or thuricin CD (31), contain l- and d-configurated thioether bonds, the latter being formed by C_α_ atom configuration–inversion during catalysis. To establish the stereochemistry of the thioether bridges, we further purified the C1_MC1_ peptide by HPLC to remove contaminating MC1 and trace amounts of unmodified C1. After purification, the C1_MC1_ peptide was subjected to deuterated hydrochloric acid (DCl) hydrolysis and amino acid derivatization with N_α_-(2,4-dinitro-5-fluorophenyl)-l-valinamide, and its amino acid content was analyzed by LC-MS as described previously (32). With this procedure, we could identify d-amino acid residues and determine whether they originated from the peptide backbone (unlabeled residues) or were produced during acid hydrolysis (incorporation of one deuterium atom). Indeed, it is well-known that, during peptide hydrolysis, free l-amino acids can spontaneously epimerize. Among the four residues involved in thioether bridges (*i.e.* Ala, Asn, Arg, and Lys), we only identified l-amino acid residues (Fig. S6), supporting that the four thioether bridges involve l-configurated amino acid residues.

### Biological activity of RumC1

**Figure 6.**
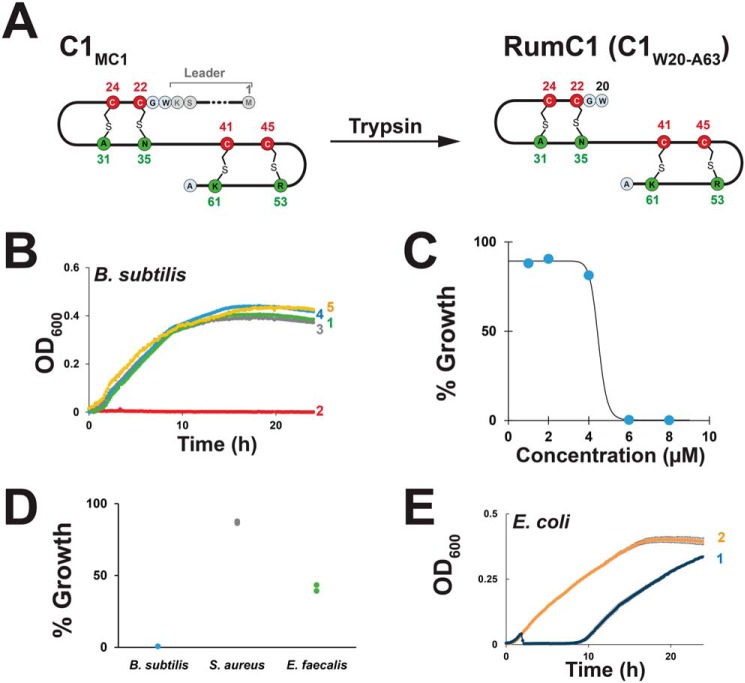
**Antimicrobial activity of RumC1.** *A*, proposed structure of RumC1 after trypsin hydrolysis of the C1_MC1_ peptide. *B*, activity assay against *B. subtilis*. Shown are growth curves of *B. subtilis* strain WT168 in the presence of 10 μm of C1_MC1_ (*1*), 10 μm of C1_MC1_ after treatment with trypsin (RumC1, *2*), 10 μm C2 (*3*), 10 μm C2 after treatment with trypsin (*4*), or without peptide (*5*). Experiments were performed in duplicate, and mean ± S.E. is indicated. *C*, activity assay against *B. subtilis* with different concentrations of RumC1. Experiments were performed in duplicate, and mean ± S.E. is indicated. *D*, activity of RumC1 against Gram-positive bacteria. *B. subtilis*, *S. aureus*, and *E. faecalis* were incubated with 70 μm RumC1. Experiments were performed in duplicate, and each data point is indicated. 100% corresponds to the growth of the bacterium in the absence of peptide. *E*, growth curves of *E. coli* strain K12 in the presence of 70 μm C1_MC1_ treated with trypsin (*1*) or in the absence of peptide (*2*). Experiments were performed in duplicate, and mean ± S.E. is indicated.

RumC was originally identified as a trypsin-dependent substance produced by *R. gnavus* and active against the Gram-positive bacteria *Clostridium perfringens* and *Bacillus subtilis* (33). This substance has been shown to be produced *in vivo* in the digestive tract of germ-free rats colonized by *R. gnavus* under the dependence of protease activity. To determine the active form of RumC, we took advantage of the C2 and C1_MC1_ peptides, which we were able to recombinantly produce in significant amounts. Preliminary assay against *C. perfringens* ATCC 13124 (22) revealed that C1_MC1_ was inactive. We thus treated C1_MC1_ with trypsin to mimic the activation process reported in previous studies (12, 33). Following trypsin hydrolysis, LC-MS analysis showed that C1_MC1_ was truncated after Lys^19^, leading to formation of a peptide ([M+4H]^4+^_obs_ 1080.98) encompassing residues Trp^20^ to Ala^63^ and containing the four thioether bridges (Fig. 6*A*). This peptide, called RumC1, exerted antimicrobial activity toward *C. perfringens*, supporting this structure being the active form (data not shown).

To better characterize the antimicrobial properties of RumC1, we used *B. subtilis*, which is a nonpathogenic and aerobic bacterium sensitive to RumC (33). Similarly to *C. perfringens*, *B. subtilis* was sensitive to RumC1 but not to C1_MC1_ (Fig. 6*B*). As an additional control, the C2 peptide, with or without trypsin treatment, was assayed against *B. subtilis* and proved to be inactive. This result supports that, in addition to removal of the leader peptide, the presence of the thioether bridges is mandatory for activity, although subtle differences between the sequences of the C1 and C2 peptides might also account for the lack of activity of the C2 peptide. The minimum inhibitory concentration of RumC1 against *B. subtilis* was determined to be 6 μm (Fig. 6*C*), a value likely to be overestimated considering the contamination of the RumC1 peptide with the radical SAM enzyme MC1. Assayed against other Gram-positive bacteria, RumC1 (70 μm) was moderately active against *Enterococcus faecalis* (Fig. 6*D*) but not against *Staphylococcus aureus*. Interestingly, assayed against *E. coli* at an identical concentration, RumC1 induced a lag phase, suggesting possible action toward Gram-negative bacteria (Fig. 6*E*). This last result was consistent with a recent study indicating possible activity of a peptide extract containing RumC against *Salmonella enteridis* (12).

## Discussion

Early studies have shown that *R. gnavus*, a commensal bacterium from the human microbiota (34), produces various anti-clostridial substances. The first substance identified was called Ruminococcin A (RumA) and proved to be a lanthibiotic (10, 35) characterized by the presence of three β-thioether bridges (13). Intriguingly this lanthipeptide was produced only when trypsin was added to the bacterial growth medium (35). Later, it was shown that *R. gnavus* produces an additional anti-clostridial substance when it colonizes the gastrointestinal tract of mono-associated rats (*i.e.* germ-free rats colonized with *R. gnavus* only) (12). Production of this elusive substance, called Ruminococcin C (RumC), was shown to be dependent on an operon notably encoding two putative radical SAM enzymes and five potential peptide precursors (C1-C5) (Fig. 1*A*) and to require trypsin for activity (11, 33). Radical SAM enzymes have been shown to introduce a broad range of posttranslational modifications in RiPPs (5, 16), including methylation (36, 37), epimerization (32, 38, 39), and carbon–carbon (40, 41) and thioether bonds (18, 25, 30, 42). Our results support that the C1 and C2 peptides from the RumC operon are modified by the radical SAM enzymes MC1 and MC2, respectively. These enzymes introduce posttranslational modifications on Ala^31^, Asn^35^, Arg^53^, and Lys^61^ in the C1 peptide (Fig. 1*E*) and on Glu^31^, Asn^35^, Arg^53^, and Arg^61^ in the C2 peptide (Fig. 2*B*). Thus, both enzymes introduce posttranslational modifications at the same locations despite involving different amino acid residues. In addition, our results demonstrate that both MC1 and MC2 can modify the same substrate (*i.e.* C1 peptide), leading to formation of an identical product (Fig. 2*D*). Collectively, these data support that the complex RumC biosynthetic operon might result from gene duplication and rearrangement events. The thuricin CD (31) and thurincin H (43) biosynthetic operons share similar features. However, although the thurincin H biosynthetic operon encodes for one radical SAM enzyme and three identical peptide precursors (31), the thuricin CD operon encodes for two radical SAM enzymes and leads to production of two sactipeptides with synergistic antimicrobial activities (43).

High-resolution LC-MS/MS analysis of C1 and C2 peptides after modification by MC1 or MC2 showed that both peptides contain α,β-dehydro-amino acid residues, the hallmark of Cα-thioether bonds. Indeed, although radical SAM enzymes have been shown recently to be able to introduce α-, β-, and γ-thioether bonds in RiPPs (18, 26, 27, 30), because of their lower stability, only S-Cα thioether bonds open during LC-MS/MS analysis, with concomitant formation of characteristic dehydro-amino acid residues (26).

Our results unambiguously establish that Cys^41^ and Cys^45^ are connected to residues 53 and 61 (*i.e.* Arg^53^ and Lys^61^ in C1 and Arg^53^ and Arg^61^ in C2, respectively), defining a C-terminal hairpin domain (Fig. 3). Regarding the N-terminal domain, it was more challenging to determine the connectivity of Cys^22^ and Cys^24^. Indeed, when we assayed peptides mutated for each residue (*i.e.* C2_A24_ and C1_A22_), a thioether bridge involving the residue in position 31 was always formed. This is likely due to the close proximity between Cys^22^ and Cys^24^, which can react with the residue in position 31 after its radical activation. We currently favor for the structure of RumC, a model based on two symmetrical hairpin domains, as shown in Fig. 6*A*, although an alternate model with Cys^22^ connected to Ala^31^ and Cys^24^ to Asn^35^ cannot be ruled out completely.

Our data also support that formation of the thioether bridges in RumC follows a processive order with an N-to-C directionality and that the N- and C-terminal domains are processed independently. Indeed, using either full-length or truncated peptides (Figs. 3 and 4), we have shown that the formation of the thioether bridge involving Cys^41^ is under the dependence of the Cys^45^-Arg^53^ bridge, supporting that Arg^53^ must be modified before Arg^61^. Of note, the efficient modification of a truncated peptide (*i.e.* the C2_28–63_ peptide) by the MC2 enzyme demonstrates that its activity is leader peptide–independent, a trend encountered in a growing number of radical SAM enzymes catalyzing RiPP posttranslational modifications (18, 32, 39, 44).

Finally, we demonstrate that trypsin treatment, which removes the leader peptide (*i.e.* residues 1–20), is mandatory to obtain a functional RumC with antibacterial activity. This treatment, which mimics the activation process suggested in early studies (33, 35), is likely to occur in the digestive tract, where RumC exerts its physiological activity. The antimicrobial properties of RumC against Gram-positive *E. faecalis*, *B. subtilis*, and Gram-negative *E. coli* bacteria suggest that RumC may target other components than the cell envelope for its antimicrobial activity. However, further studies will be required to decipher its mode of action.

In conclusion, we have deciphered the structure of RumC, a sactipeptide containing four Cα-thioether bridges in the L configuration. Although sactipeptides with up to four thioether bridges, including thurincin H (28) and huazacin (26), have been reported, the structure of RumC is unique, as it is based on two hairpin domains. Indeed, all sactipeptides described so far are folded as a single hairpin domain with thioether bridges linking cysteine residues from the N-terminal domain to residues from the C-terminal domain. Our data also support that, in RumC, installation of the thioether bridges follows a precisely defined order suggestive of a processive mode of action. Indeed, although a growing number of radical SAM enzymes have been shown to be involved in the biosynthesis of S-Cα–linked (18, 28, 30, 31, 45, 46), S-Cβ–linked, and S-Cγ–linked thioether-containing peptides (26), it is currently unknown whether the formation of these bridges is random. We have shown here that MC2, similarly to the radical SAM epimerase PoyD (32), introduces modifications in a sequential order. However, contrary to PoyD, MC2 has an N-to-C directionality. Finally, we have established that the presence of thioether bridges and removal of the leader peptide are both required for the antimicrobial activity of RumC. Although several hypotheses have been proposed for this dependence, our data support that the leader peptide impedes the biological activity of RumC. Further investigations regarding the other precursor peptides (*i.e.* C3 to C5) should also clarify whether these peptides, after posttranslational modification, have similar activity than RumC1 or exhibit synergistic properties, as reported for thuricin CD. RumC thus delineates a novel class of sactipeptides and is the first member of this natural product family to be isolated from the human microbiota. Intriguingly, by targeting Gram-positive and Gram-negative bacteria, it could contributes to shape this unique and complex ecosystem (6).

## Experimental procedures

### Cloning, expression, and purification of C1 and C2 peptides

The *c1* and *c2* genes were synthesized by Life Technologies (Thermo Fisher GeneArt®) and ligated in the pRSFDuet-1 plasmid with a His_6_ tag fusion and transformed in *E. coli* BL21 (DE3) (Life Technologies). Peptide production was performed in Luria-Bertani, and cells were harvested after 20 h by centrifugation (5500 rpm, 10 min at 4 °C). Cells were suspended in buffer A (50 mm Tris and 300 mm KCl (pH 7.5)) supplemented with 1% v/v Triton X-100. Cells were disrupted by sonication, followed by ultracentrifugation (45,000 rpm for 1.5 h at 4 °C) to remove cell debris. The supernatant was loaded on Ni-NTA Fast Flow Gel (Qiagen) equilibrated previously with buffer A. The Ni-NTA gel was washed successively with buffer A containing 25 mm and 75 mm imidazole. The peptide was eluted with buffer A containing 500 mm imidazole. This fraction was loaded on an NAP10 column equilibrated previously with buffer A. The peptide was concentrated in an Amicon concentrator (molecular weight cutoff of 3 kDa, Millipore) and stored at −80 °C. Peptide purity was assessed by SDS-PAGE (18% (w/v)), and the concentration was determined using a NanoDrop spectrophotometer.

### Cloning, expression, and purification of C1 and C2 coexpressed with MC1 and MC2

Plasmids pRSFDuet-His-C1 and pRSFDuet-His-C2 were used as a template. The plasmids were digested with NdeI/XhoI, and the *mc1* and *mc2* genes (synthesized by Life Technologies) were ligated into the respective construct. Protein expression and purification were performed as described above. Purity was assessed by SDS-PAGE and LC-MS analysis. Peptide concentration was determined by NanoDrop spectrophotometer.

### Cloning, expression, and purification of the radical SAM enzyme MC2

The *RumMC2* gene was synthesized by Life Technologies and ligated in the pET-28a(+) plasmid. After sequencing, the construction was transformed in *E. coli* BL21 (DE3) star cells (Life Technologies). Protein expression and purification were performed as described above. Proteins were concentrated in an Amicon concentrator (molecular weight cutoff of 10 kDa, Millipore) and stored at −80 °C. 12% (w/v) SDS-PAGE was run to confirm the purity of the protein, and concentration was determined by NanoDrop spectrophotometer.

### Cloning, expression, and purification of the C2_A22-A24_, C2_A31-A35_, and C2_A24_ mutants

The mutants were obtained by site-directed mutagenesis. His-C2-RSFDuet was used as a template with the following primers: 5′-CAAAGGTGGTGCTAAAGCTAGCGGTGGTG-3′ and 5′-CACCACCGCTAGCTTTAGCACCACCTTTG-3′ to mutate Cys^22^ and Cys^24^ to alanine. Primers 5′-CGGCATATGCTGTTGGTTATGCTGGTAATAA-3′ and 5′-TTATTACCAGCATAACCAACAGCATATGCCG-3′ were used to mutate Cys^41^ and Cys^45^ to alanine. Primers 5′-CAAAGGTGGTTGTAAAGCTAGCGGTGGTG-3′ and 5′-CACCACCGCTAGCTTTACAACCACCTTTG-3′ were used to mutate Cys^24^ to alanine. For each reaction, the PCR mix contained 5% (v/v) of DMSO, 200 nm each primer, 100 ng of DNA template, 200 μm dNTP, and 1 μl of PfuUltra II Fusion HS DNA polymerase (Agilent). The PCR cycling parameters used were as follows: 95 °C for 2 min, followed by 30 cycles of 95 °C for 30 s, 50 °C for 30 s, and 72 °C for 150 s. After digestion with 20 units of DpnI (37 °C, 1 h), the PCR product was used to transform TOP10 *E. coli* cells. Final constructs were transformed in *E. coli* BL21 (DE3) cells (Life Technologies). Expression and purification of the peptides were performed as detailed above.

### Peptide synthesis

Peptides were synthesized by Proteogenix and resuspended in 100% (v/v) DMSO. Peptides were as follows: C2_28–63_, AVVENSHNAGPAYCVGYCGNNGVVTRNANANLARTK; C2_28–63_A41, AVVENSHNAGPAYAVGYCGNNGVVTRNANANLARTK; C2_28–63_A45, AVVENSHNAGPAYCVGYAGNNGVVTRNANANLARTK; and C2_28–63_A41A45, AVVENSHNAGPAYAVGYAGNNGVVTRNANANLARTK.

### Iron–sulfur cluster reconstitution

Protein reconstitution was performed under anaerobic conditions at 4 °C using a 12 molar excess of NH_4_)_2_Fe(SO_4_)_2_ (Sigma-Aldrich) and Na_2_S (Sigma-Aldrich). The excess of unbound iron and sulfur was removed by Sephadex G25 column (GE Healthcare) equilibrated with buffer A. Proteins were concentrated using an Amicon concentrator (molecular weight cutoff of 10 kDa, Millipore).

### Enzymatic assays

All assays were performed in an anaerobic chamber. Freshly reconstituted protein was used to perform the assays. Protein was concentrated at 200 μm and incubated with 1 mm peptide. Reactions were quenched by adding 0.1% (v/v) of formic acid for LC-MS analysis.

### HPLC analysis and purification

Peptides were analyzed and purified using a Zorbax Eclipse Plus C18 Rapid Resolution HT (2 × 50 mm, 1.8 μm, 100 Å, Agilent) by loading 10–20 μl of each sample diluted 10 times in 0.1% (v/v) formic acid. Elution was performed at a flow rate of 0.3 ml/min using an acetonitrile gradient between 10% to 30% (v/v) of acetonitrile 80% (v/v), formic acid 0.1% (v/v). Peptide UV detection was performed at 215 nm.

### LC-MS analysis

Each peptide was analyzed by LC-MS using a Q Exactive Focus mass spectrometer (Thermo Fisher Scientific). Peptide separation was performed on Ultimate 3000 nanoHPLC and Vanquish ultra-high-performance liquid chromatography systems. The C1, C1_MC1_, and C2_MC2_ peptides were analyzed using a Proswift RP4H (Thermo Fisher) monolithic nanocolumn (0.1 × 250 mm), and the C2_MC1_ and C1_MC2_ peptides were analyzed using a Zorbax Eclipse Plus C18 Rapid Resolution HT column (2 × 50 mm, 1.8 μm, 95 Å). Acetonitrile gradients between 10%–30% and 15%–25% in formic acid (0.1%) were used. Mass analysis was performed at a resolution of 35,000 (*m/z*, 200) with a MS range of 500–1300 and MS/MS analysis. The collision energy was optimized for each peptide (between 22% and 25%) to reduce formation of internal fragments. The lock mass option was activated to enhance mass accuracy. For each peptide, several scans (between 5 and 10) were merged to enhance the quality of the data. Data were deconvoluted using Xtract tools included in the Freestyle software suite, version 1.3 (Thermo Electron). All daughter ions observed were verified and annotated manually.

### Determination of thioether bridge configuration

The C1_MC1_ peptide was purified by HPLC and dried using a centrifugal vacuum concentrator. Hydrolysis was performed in DCl (6 n) under vacuum conditions at 110 °C for 12 h. Reaction mixtures were incubated for 1 h at 42 °C after addition of 4 μl of NaHCO_3_ 1 m, 3 μl of *N*-α-(2,4-dinitro-5-fluorophenyl)-l-valinamide at 10 mg/ml, and 13 μl of H_2_O. Final mixtures were diluted in 0.1% (v/v) formic acid before LC-MS/MS analysis as described previously (32).

### Antimicrobial assay

Peptide purity was assessed by LC-MS/MS and HPLC analysis. Each peptide was analyzed by data-dependent top-down analysis MS/MS fragmentation analysis on a Q-exactive Focus mass spectrometer. Peptide digestion was performed using trypsin (1% (v/v)) at 37 °C for 12 h. Growth experiments were performed using M17 (Glucose 1% (v/v)) medium for *E. faecalis* and Mueller–Hinton broth for *E. coli* (K12), *B. subtilis* 168, and *S. aureus*, respectively. An overnight culture was used to inoculated the growth medium at a final *A*_600_ = 0.1. After 5 h of growth, a fresh culture was prepared (*A*_600_ = 0.05) for the antimicrobial assay. 10 μl of sample was added to 90 μl of inoculated medium. Growth analysis was performed using a Bioscreen apparatus for 24 h at 37 °C.

## Author contributions

C. Balty, A. G., A. B., and O. B. formal analysis; C. Balty, A. G., A. B., and O. B. investigation; C. Balty, A. G., L. F., C. Brewee, M. B., X. K., A. B., and O. B. methodology; A. B. and O. B. conceptualization; A. B. and O. B. supervision; A. B. and O. B. funding acquisition; A. B. and O. B. validation; A. B. and O. B. writing-original draft; A. B. and O. B. project administration; A. B. and O. B. writing-review and editing.

## Supplementary Material

Supporting Information

## Footnotes

3 The abbreviations used are:

RiPP: ribosomally synthesized and posttranslationally modified peptide
Ni-NTA: nickel-nitrilotriacetic acid.
