# Supplementary material for: Ruminococcin C, an anti-clostridial sactipeptide produced by a prominent member of the human microbiota Ruminococcus gnavus
Source: J Biol Chem. 2019 Jul 23;294(40):14512–25. doi: 10.1074/jbc.RA119.009416 (PMC6779426; doi:10.1074/jbc.RA119.009416)
Supplement: Supporting Information [file supp_294_40_14512__index.html]

Ruminococcin C, an anti-clostridial sactipeptide produced by a prominent member of the human microbiota Ruminococcus gnavus — Ruminococcin C, novel sactipeptide from the human microbiota — Ruminococcin C, an anti-clostridial sactipeptide produced by a prominent member of the human microbiota Ruminococcus gnavus — Ruminococcin C, a new sactipeptide from the human microbiota — Supporting Information 

# Ruminococcin C, an anti-clostridial sactipeptide produced by a prominent member of the human microbiota *Ruminococcus gnavus*

## Supporting Information

- Supporting Information (to be published online) - Supporting information
