# Supplementary material for: Ruminococcin C, an anti-clostridial sactipeptide produced by a prominent member of the human microbiota Ruminococcus gnavus
Source: J Biol Chem. 2019 Jul 23;294(40):14512–25. doi: 10.1074/jbc.RA119.009416 (PMC6779426; doi:10.1074/jbc.RA119.009416)
Supplement: Supporting Information [file supp_RA119.009416_152933_1_supp_365613_pywync.pdf]

Supporting Information for:

Ruminococcin C, an anti-clostridial sactipeptide produced by a prominent member of the human microbiota *Ruminococcus gnavus*

Clémence Balty[1], Alain Guillot[1], Laura Fradale[1], Clémence Brewee[1], Mylène Boulay[1], Xavier Kubiak[1], Alhosna Benjdia[1]* & Olivier Berteau[1]*

[1]Micalis Institute, ChemSyBio, INRA, AgroParisTech, Université Paris-Saclay, 78350 Jouy-en-Josas, France

* : Corresponding authors

.Figure S1- Sequence of the peptides C1 to C5 .... **2**

Figure S2 - Sequence alignment of the radical SAM enzymes MC1 and MC2. .... **3**

Figure S3 – LC-MS analysis of the C2 peptide expressed in *E. coli* **4**

Figure S4 – LC-MS analysis of the $C2_{A22\text{-}A24}$ peptide **5**

Figure S5 - LC-MS analysis of the $C1_{A22}$ peptide **6**

Figure S6 - LC-MS analysis of the amino acid content of peptide $C1_{MC1}$ **7**

Table S1 – Peptides used in this study and their theoretical and observed (obs) masses **8**

Table S2 - Theoretical Mass fragments of peptides C1 and C2 .... **9**

Table S3 - Theoretical Mass fragments of peptides $C2_{A22\text{-}A24}$ and $C2_{A41\text{-}A45}$ **10**

Table S4 - Theoretical Mass fragments of peptides $C2_{A24}$ and $C1_{A22}$ **11**

Table S5 - Theoretical Mass fragments of peptides $C2_{28\text{-}63}$ $[M+H]^+$ and $C2_{28\text{-}63}$ A41 $[M+H]^+$ **12**

A

```
C1   MRKIVAGKLQTGADFEGSKWGCVCSGSTAVANSHNAGPAYCVGYCGNNGVVTRNANANVAKTA
C2   MRKIVAGKLQTGADFEGSKGGCKCSGGAVVENSHNAGPAYCVGYCGNNGVVTRNANANLARTK
C3   MKLVETKTTKTGTNFEGNRAGCICNGTVAVANSHNAGPAYCVGYCGNSGVVTRNANANVAKTA
C4   MRLVQSKRIATGFNFEGSKAGCVCSGTVAVANSHNAGPAYCVGYCGNNGEVTRNANYNIARRS
C5   MKLVTSKTMKTGTNFEGNKAGCICSGSVAVANSHNAGPAYCVGYCGNNGAVTRNANANLARTA
```

B

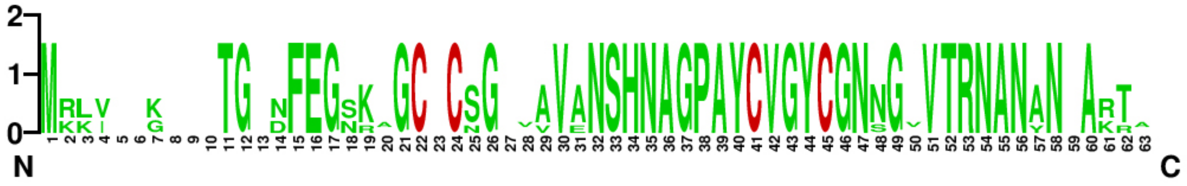

Figure S1- Sequence of the peptides C1 to C5

(A) Sequence of the predicted peptide C1 to C5. Identical residues are in red and conserved cysteine residues are highlighted in red. (B) Logo representation of the conserved amino acid residues in peptide C1 to C5. The overall height represents the sequence conservation for each position.

```
MC1      MKEHLKKIKETIDVERGYSLGTVFTTRNKQYMYDTGTGKVFECGVNEYRILKSLFEQTSLPDKVD
MC2    MKAKEHLKKIKGTIDVERGYSLGTVFTTRNKQYMYDTGTGKVFECGANEYQILKSLFEQTSLPEKVE

MC1    GVSEEELEAAYRNIWEMVEAEHILQVSPNLKFVRETDETLRDLLRYDLQQVILELTEQCNMRCRYCI
MC2    GSSEEELEAAYRNIWEMIETEHILQISPDLKFVKETDETLRDLLRYDLQQVILELTEQCNMRCRYCI

MC1    YNEHNEGYRNFSPKAMTWDVAKRAVEYARDNSGDKVAISFYGGEPLVQFELMKKTIDYSRQIIKGKE
MC2    YNEHNEGYRNFSPKAMTWEVAKRAVEYARDNSGDKVAVSFYGGEPLVQFELMKKTIDYSRQIIKGKE

MC1    LTFSFSTNLTLVTPEIAAYVAGVEGMSVLASIDGPEGIHDAYRVMSGGKGSFEKAIQGLKYLVEAFG
MC2    LTFSFSTNLTLVTPEIAAYVAGVEGMSVLASIDGPEKIHDAYRVMSGGKGSFGKAIQGLKYLVEAFG

MC1    ERAKESIVINTVVCPPFSAKKLDAIKEFFEGLSWLPKEMVKKCDYVEYGSVREEDISMEYAGDGEFI
MC2    ERAKESIVINTVVCPPFSAKKLDAIKEFFEGLNWLPKEMVKKCDYVEYGSVREEDISMEYAGDGEFV

MC1    GEELDGFTLDAIEGWALARDLEEEDPKSYVAGIVADKLVRIHNRRQTQEPCKDLRRNGCCIPGNRRV
MC2    GEELDGFTLDAIEGWALTRDLEEQDPKSYVAGIVTDKLVRIHNRRQTQEPCKDLRRNGCCIPGNRRV

MC1    YVKTDGKFLLCEKTGDAPDIGNVFEGADLEKIKKYYIEEYDEKSITRCNECWARNLCGLCYAACYEA
MC2    YVKADGKFLLCEKTGDAPDIGNVFEGADLEKIKKYYIEEYDEKSLTRCNECWARNLCGLCYAACYEA

MC1    EGIDMERKEKVCGAHRYATKGELISYYSILEEKPEVIEEIDAVPYY
MC2    EGIDMERKEKVCGAHRYATKGELISYYSILEEKPEVIEEIDAVPYY
```

Figure S2 - Sequence alignment of the radical SAM enzymes MC1 and MC2.
Identical residues are in red and conserved cysteines residues are highlighted.
(Identity 95%, similarity 97.5%).

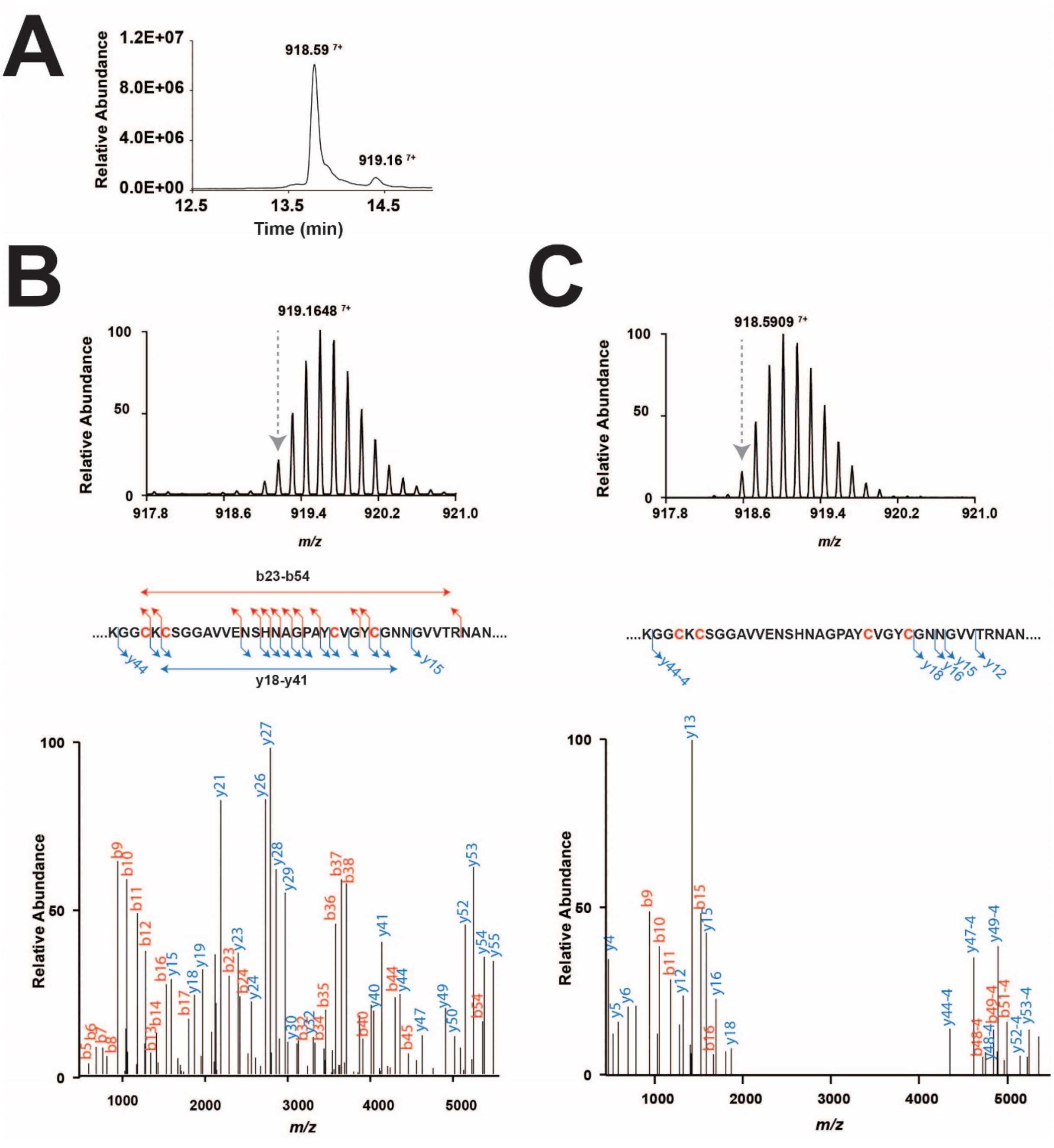

Figure S3 – LC-MS analysis of the C2 peptide expressed in *E. coli*.

(**A**) LC-MS profile of C2 peptide. **(B**) MS (top panel) and LC MS/MS (lower panel) analysis of the linear form of **C2** peptide. (**C**) MS (top panel) and LC MS/MS (lower panel) analysis of the oxidized form of **C2** peptide.

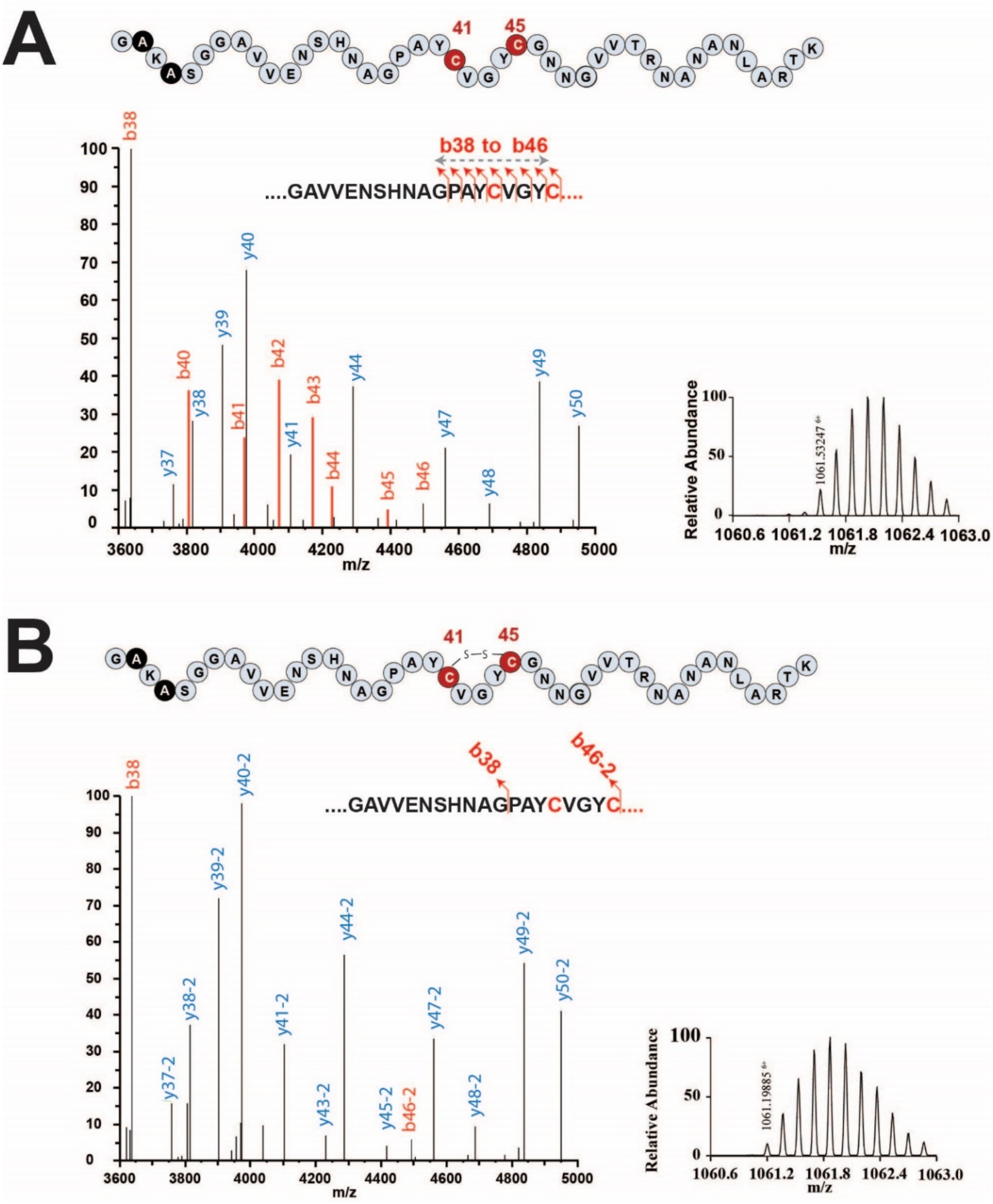

Figure S4 – LC-MS analysis of the $C2_{A22\text{-}A24}$ peptide

(**A**) LC-MS/MS analysis (left panel) and MS spectrum (right panel) of the $C2_{A22\text{-}A24}$ peptide. The peptide sequence is indicated above the panel. (**B**) LC-MS/MS analysis (left panel) and MS spectra (right panel) of $C2_{A22\text{-}A24}$ ox peptide. Representation of the peptide with one disulfide bond ($C2_{A22\text{-}A24}$ ox) is indicated above the panel.

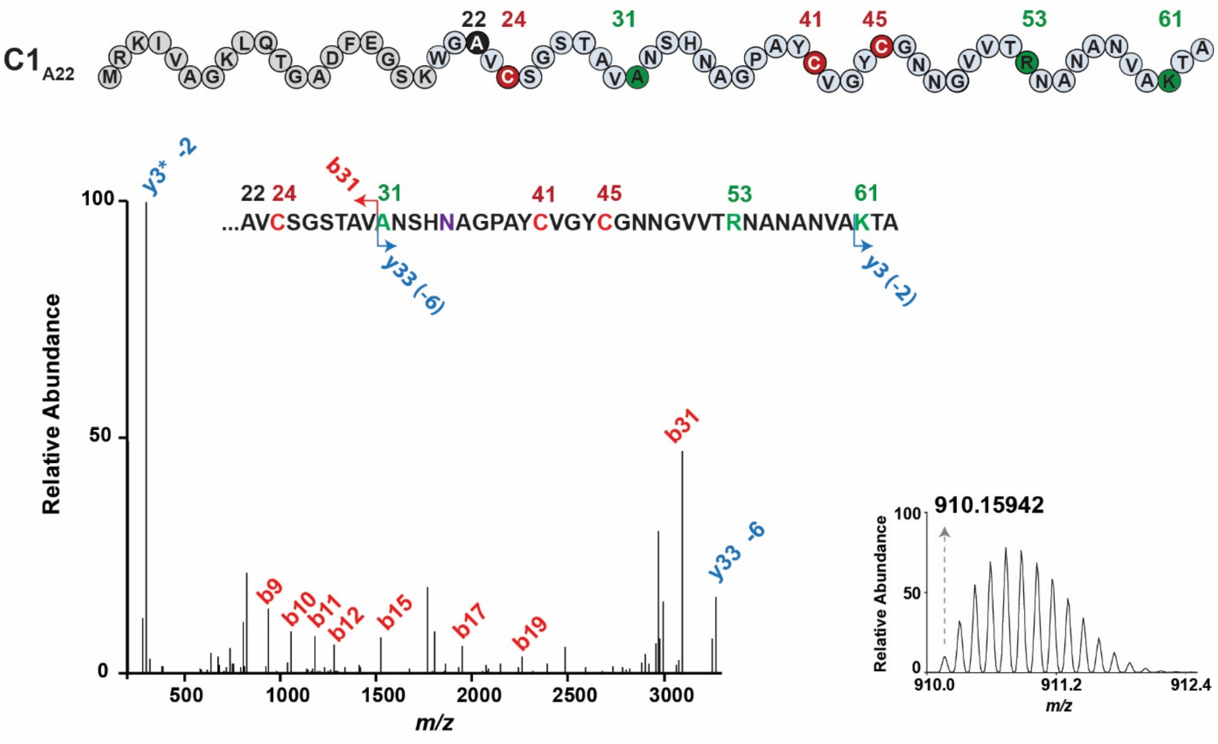

Figure S5 - LC-MS analysis of the C1A22 peptide

LC-MS/MS analysis (left panel) and MS spectrum (right panel) of the **C1A22** peptide. The peptide sequence is indicated above the panel.

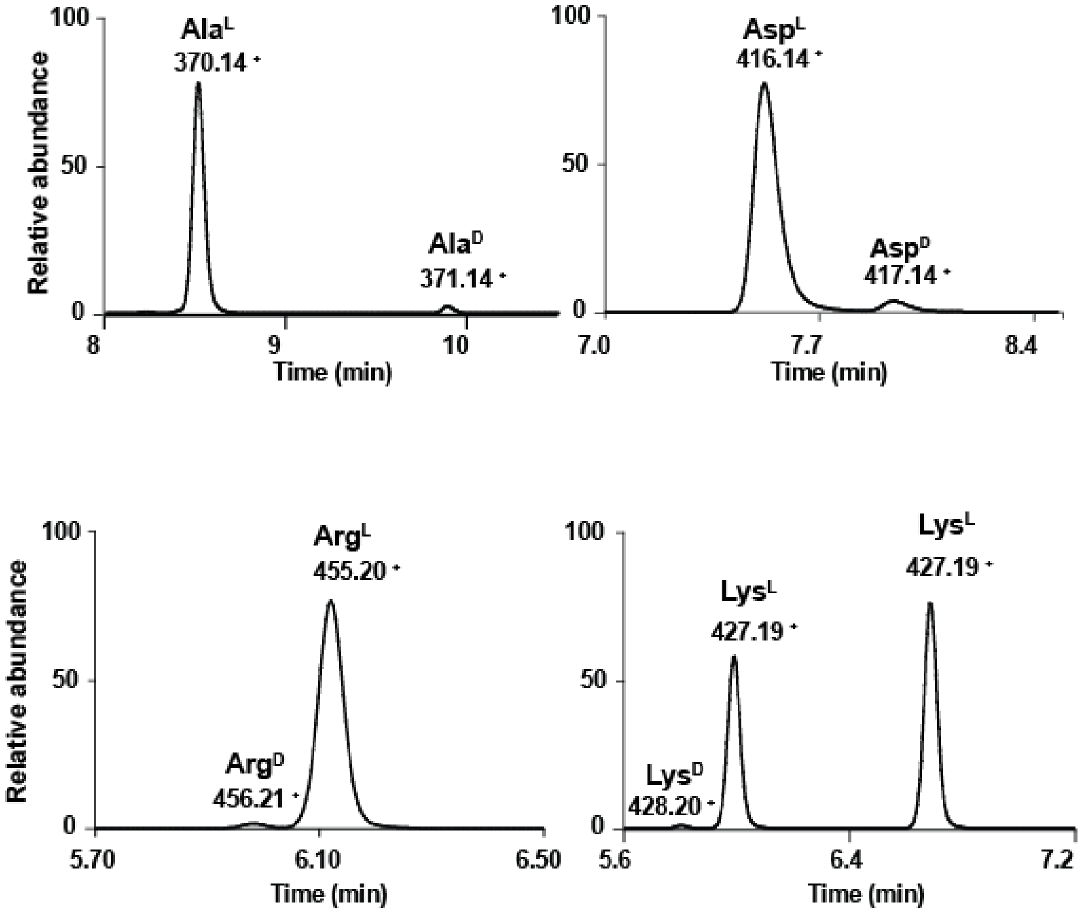

Figure S6 - LC-MS analysis of the amino acid content of peptide C1$_{MC1}$

After purification, C1 was subjected to acid hydrolysis (DCl 6M, 110°C, 12 hours), amino acids were derivatized using N_{α}-(2,4-dinitro-5-fluorophenyl)-L-valinamide and analyzed by LC-MS. During acid hydrolysis Asn is converted in Asp. Theoretical molecular weights: L-Ala: $[M+H]^+$ = 370.14; L-Asp: $[M+H]^+$ = 416.14; L-Arg: $[M+H]^+$ = 455.21 and L-Lys $[M+H]^+$ = 427.19 (L-Lys appears as two peaks corresponding to the derivatization of the NH_2 on the side chain or on the main chain). The +1 Da mass increment in D-Ala, D-Asp, D-Arg and D- Lys indicates deuterium incorporation during acid hydrolysis.

Table S1 – Peptides used in this study and their theoretical and observed (obs) masses

Peptide	Formula	$[M+7H]^{7+}$	$[M+7H]^{7+}_{obs}$
**C1 linear form**	C269 H429 N85 O87 S5	915.5839	-
**C1 oxidized form**	C269 H425 N85 O87 S5	915.0080	915.0098
**C1 (-8H)**	C269 H421 N85 O87 S5	914.4321	914.4381
**C2 linear form**	C267 H436 N88 O87 S5	919.1644	919.1637
**C2 oxidized form**	C267 H432 N88 O87 S5	918.58854	918.5896
**C2 (-8H)**	C267 H428 N88 O87 S5	918.01264	918.0131

Peptide	Formula	$[M+6H]^{6+}$	$[M+6H]^{6+}_{obs}$	$[M+6H]^{6+}$ (-4H)	$[M+6H]^{6+}_{obs}$ (-4H)
**$C2_{A22-A24}$**	C267 H436 N88 O87 S3	1061.5333	1061.5327	1060.8614	1060.862
**$C2_{A41-A45}$**	C267 H436 N88 O87 S3	1061.5333	1061.5325	1060.8614	1060.8613

Peptide	Formula	$[M+6H]^{6+}$	$[M+6H]^{6+}_{obs}$	$[M+6H]^{6+}$ (-6H)	$[M+6H]^{6+}_{obs}$ (-6H)
**$C2_{A24}$ oxidized form**	C267 H436 N88 O87 S4	1066.5260	1066.5284	1065.8541	1065.8558
**Peptide**	**Formula**	$[M+7H]^{7+}$	$[M+7H]^{7+}_{obs}$	$[M+7H]^{7+}$ (-6H)	$[M+7H]^{7+}_{obs}$ (-6H)
**$C1_{A22}$**	C269 H429 N85 O87 S4	911.0164	-	910.1526	910.1594

Peptide	Formula	$[M+4H]^{4+}$	$[M+4H]^{4+}_{obs}$	$[M+4H]^{4+}$ (-4H)	$[M+4H]^{4+}$ (-4H)
**$C2_{28-63}$**	C154 H247 N52 O51 S2	926.9497	926.9525	926.1938	925.9474

Peptides	Formula	$[M+4H]^{4+}$	$[M+4H]^{4+}_{obs}$	$[M+4H]^{4+}$ (-2H)	$[M+4H]^{4+}_{obs}$ (-2H)
**$C2_{28-63}$ A45**	C154 H246 N52 O51 S	918.9567	918.9636	-	-
**$C2_{28-63}$ A41**	C154 H246 N52 O51 S	918.9567	918.9595	918.4527	918.4591

Peptide	Formula	$[M+7H]^{7+}$	$[M+7H]^{7+}_{obs}$
**C2 species B**	C267 H432 N88 O87 S5	918.58854	918.5877
**C2 species C**	C267 H432 N88 O87 S5	918.58854	918.5882

Peptide	Formula	$[M+4H]^{4+}$	$[M+4H]^{4+}_{obs}$
**$C1_{MC1}$-W20-A63**	C179 H278 N58 O60 S4	1080.9758	1080.9807

Table S2 - Theoretical Mass fragments of peptides C1 and C2

C1

		b	y	
G	1	58.0288	6403.0435	64
M	2	189.0693	6346.0220	63
R	3	345.1704	6214.9815	62
K	4	473.2653	6058.8804	61
I	5	586.3494	5930.7854	60
V	6	685.4178	5817.7014	59
A	7	756.4549	5718.6330	58
G	8	813.4764	5647.5959	57
K	9	941.5714	5590.5744	56
L	10	1054.6554	5462.4794	55
Q	11	1182.7140	5349.3954	54
T	12	1283.7617	5221.3368	53
G	13	1340.7831	5120.2891	52
A	14	1411.8203	5063.2676	51
D	15	1526.8472	4992.2305	50
F	16	1673.9156	4877.2036	49
E	17	1802.9582	4730.1352	48
G	18	1859.9797	4601.0926	47
S	19	1947.0117	4544.0711	46
K	20	2075.1067	4457.0391	45
W	21	2261.1860	4328.9441	44
G	22	2318.2074	4142.8648	43
C	23	2421.2166	4085.8434	42
V	24	2520.2850	3982.8342	41
C	25	2623.2942	3883.7658	40
S	26	2710.3263	3780.7566	39
G	27	2767.3477	3693.7245	38
S	28	2854.3797	3636.7031	37
T	29	2955.4274	3549.6711	36
A	30	3026.4645	3448.6234	35
V	31	3125.5330	3377.5863	34
A	32	3196.5701	3278.5178	33
N	33	3310.6130	3207.4807	32
S	34	3397.6450	3093.4378	31
H	35	3534.7039	3006.4058	30
N	36	3648.7469	2869.3469	29
A	37	3719.7840	2755.3039	28
G	38	3776.8054	2684.2668	27
P	39	3873.8582	2627.2454	26
A	40	3944.8953	2530.1926	25
Y	41	4107.9586	2459.1555	24
C	42	4210.9678	2296.0922	23
V	43	4310.0362	2193.0830	22
G	44	4367.0577	2094.0146	21
Y	45	4530.1210	2036.9931	20
C	46	4633.1302	1873.9298	19
G	47	4690.1517	1770.9206	18
N	48	4804.1946	1713.8991	17
N	49	4918.2375	1599.8562	16
G	50	4975.2590	1485.8133	15
V	51	5074.3274	1428.7918	14
V	52	5173.3958	1329.7234	13
T	53	5274.4435	1230.6550	12
R	54	5430.5446	1129.6073	11
N	55	5544.5875	973.5062	10
A	56	5615.6247	859.4633	9
N	57	5729.6676	788.4261	8
A	58	5800.7047	674.3832	7
N	59	5914.7476	603.3461	6
V	60	6013.8160	489.3032	5
A	61	6084.8532	390.2348	4
K	62	6212.9481	319.1976	3
T	63	6313.9958	191.1027	2
A	64	6385.0329	90.0550	1

C2

		b	y	
G	1	58.0288	6428.1075	64
M	2	189.0693	6371.0860	63
R	3	345.1704	6240.0455	62
K	4	473.2653	6083.9444	61
I	5	586.3494	5955.8494	60
V	6	685.4178	5842.7654	59
A	7	756.4549	5743.6970	58
G	8	813.4764	5672.6599	57
K	9	941.5714	5615.6384	56
L	10	1054.6554	5487.5434	55
Q	11	1182.7140	5374.4594	54
T	12	1283.7617	5246.4008	53
G	13	1340.7831	5145.3531	52
A	14	1411.8203	5088.3316	51
D	15	1526.8472	5017.2945	50
F	16	1673.9156	4902.2676	49
E	17	1802.9582	4755.1992	48
G	18	1859.9797	4626.1566	47
S	19	1947.0117	4569.1351	46
K	20	2075.1067	4482.1031	45
G	21	2132.1281	4354.0081	44
G	22	2189.1496	4296.9867	43
C	23	2292.1588	4239.9652	42
K	24	2420.2537	4136.9560	41
C	25	2523.2629	4008.8611	40
S	26	2610.2950	3905.8519	39
G	27	2667.3164	3818.8198	38
G	28	2724.3379	3761.7984	37
A	29	2795.3750	3704.7769	36
V	30	2894.4434	3633.7398	35
V	31	2993.5118	3534.6714	34
E	32	3122.5544	3435.6030	33
N	33	3236.5973	3306.5604	32
S	34	3323.6294	3192.5175	31
H	35	3460.6883	3105.4854	30
N	36	3574.7312	2968.4265	29
A	37	3645.7683	2854.3836	28
G	38	3702.7898	2783.3465	27
P	39	3799.8426	2726.3250	26
A	40	3870.8797	2629.2722	25
Y	41	4033.9430	2558.2351	24
C	42	4136.9522	2395.1718	23
V	43	4236.0206	2292.1626	22
G	44	4293.0421	2193.0942	21
Y	45	4456.1054	2136.0727	20
C	46	4559.1146	1973.0094	19
G	47	4616.1360	1870.0002	18
N	48	4730.1790	1812.9788	17
N	49	4844.2219	1698.9358	16
G	50	4901.2433	1584.8929	15
V	51	5000.3118	1527.8714	14
V	52	5099.3802	1428.8030	13
T	53	5200.4279	1329.7346	12
R	54	5356.5290	1228.6869	11
N	55	5470.5719	1072.5858	10
A	56	5541.6090	958.5429	9
N	57	5655.6519	887.5058	8
A	58	5726.6890	773.4629	7
N	59	5840.7320	702.4257	6
L	60	5953.8160	588.3828	5
A	61	6024.8532	475.2988	4
R	62	6180.9543	404.2616	3
T	63	6282.0019	248.1605	2
K	64	6410.0969	147.1129	1

Table S3 - Theoretical Mass fragments of peptides C2$_{A22-A24}$ and C2$_{A41-A45}$

C2$_{A22-A24}$

		b	*y*	
G	1	58.0288	6364.1633	64
M	2	189.0693	6307.1419	63
R	3	345.1704	6176.1014	62
K	4	473.2653	6020.0003	61
I	5	586.3494	5891.9053	60
V	6	685.4178	5778.8212	59
A	7	756.4549	5679.7528	58
G	8	813.4764	5608.7157	57
K	9	941.5714	5551.6942	56
L	10	1054.6554	5423.5993	55
Q	11	1182.7140	5310.5152	54
T	12	1283.7617	5182.4566	53
G	13	1340.7831	5081.4090	52
A	14	1411.8203	5024.3875	51
D	15	1526.8472	4953.3504	50
F	16	1673.9156	4838.3234	49
E	17	1802.9582	4691.2550	48
G	18	1859.9797	4562.2124	47
S	19	1947.0117	4505.1910	46
K	20	2075.1067	4418.1589	45
G	21	2132.1281	4290.0640	44
G	22	2189.1496	4233.0425	43
A	23	2260.1867	4176.0211	42
K	24	2388.2817	4104.9839	41
A	25	2459.3188	3976.8890	40
S	26	2546.3508	3905.8519	39
G	27	2603.3723	3818.8198	38
G	28	2660.3937	3761.7984	37
A	29	2731.4309	3704.7769	36
V	30	2830.4993	3633.7398	35
V	31	2929.5677	3534.6714	34
E	32	3058.6103	3435.6030	33
N	33	3172.6532	3306.5604	32
S	34	3259.6852	3192.5175	31
H	35	3396.7441	3105.4854	30
N	36	3510.7871	2968.4265	29
A	37	3581.8242	2854.3836	28
G	38	3638.8456	2783.3465	27
P	39	3735.8984	2726.3250	26
A	40	3806.9355	2629.2722	25
Y	41	3969.9988	2558.2351	24
C	42	4073.0080	2395.1718	23
V	43	4172.0764	2292.1626	22
G	44	4229.0979	2193.0942	21
Y	45	4392.1612	2136.0727	20
C	46	4495.1704	1973.0094	19
G	47	4552.1919	1870.0002	18
N	48	4666.2348	1812.9788	17
N	49	4780.2777	1698.9358	16
G	50	4837.2992	1584.8929	15
V	51	4936.3676	1527.8714	14
V	52	5035.4360	1428.8030	13
T	53	5136.4837	1329.7346	12
R	54	5292.5848	1228.6869	11
N	55	5406.6277	1072.5858	10
A	56	5477.6649	958.5429	9
N	57	5591.7078	887.5058	8
A	58	5662.7449	773.4629	7
N	59	5776.7878	702.4257	6
L	60	5889.8719	588.3828	5
A	61	5960.9090	475.2988	4
R	62	6117.0101	404.2616	3
T	63	6218.0578	248.1605	2
K	64	6346.1528	147.1129	1

C2$_{A41-A45}$

		b	*y*	
G	1	58.0288	6364.1633	64
M	2	189.0693	6307.1419	63
R	3	345.1704	6176.1014	62
K	4	473.2653	6020.0003	61
I	5	586.3494	5891.9053	60
V	6	685.4178	5778.8212	59
A	7	756.4549	5679.7528	58
G	8	813.4764	5608.7157	57
K	9	941.5714	5551.6942	56
L	10	1054.6554	5423.5993	55
Q	11	1182.7140	5310.5152	54
T	12	1283.7617	5182.4566	53
G	13	1340.7831	5081.4090	52
A	14	1411.8203	5024.3875	51
D	15	1526.8472	4953.3504	50
F	16	1673.9156	4838.3234	49
E	17	1802.9582	4691.2550	48
G	18	1859.9797	4562.2124	47
S	19	1947.0117	4505.1910	46
K	20	2075.1067	4418.1589	45
G	21	2132.1281	4290.0640	44
G	22	2189.1496	4233.0425	43
C	23	2292.1588	4176.0211	42
K	24	2420.2537	4073.0119	41
C	25	2523.2629	3944.9169	40
S	26	2610.2950	3841.9077	39
G	27	2667.3164	3754.8757	38
G	28	2724.3379	3697.8542	37
A	29	2795.3750	3640.8328	36
V	30	2894.4434	3569.7957	35
V	31	2993.5118	3470.7272	34
E	32	3122.5544	3371.6588	33
N	33	3236.5973	3242.6162	32
S	34	3323.6294	3128.5733	31
H	35	3460.6883	3041.5413	30
N	36	3574.7312	2904.4824	29
A	37	3645.7683	2790.4394	28
G	38	3702.7898	2719.4023	27
P	39	3799.8426	2662.3809	26
A	40	3870.8797	2565.3281	25
Y	41	4033.9430	2494.2910	24
A	42	4104.9801	2331.2277	23
V	43	4204.0485	2260.1905	22
G	44	4261.0700	2161.1221	21
Y	45	4424.1333	2104.1007	20
A	46	4495.1704	1941.0373	19
G	47	4552.1919	1870.0002	18
N	48	4666.2348	1812.9788	17
N	49	4780.2777	1698.9358	16
G	50	4837.2992	1584.8929	15
V	51	4936.3676	1527.8714	14
V	52	5035.4360	1428.8030	13
T	53	5136.4837	1329.7346	12
R	54	5292.5848	1228.6869	11
N	55	5406.6277	1072.5858	10
A	56	5477.6649	958.5429	9
N	57	5591.7078	887.5058	8
A	58	5662.7449	773.4629	7
N	59	5776.7878	702.4257	6
L	60	5889.8719	588.3828	5
A	61	5960.9090	475.2988	4
R	62	6117.0101	404.2616	3
T	63	6218.0578	248.1605	2
K	64	6346.1528	147.1129	1

C2$_{A24}$

		b	*y*	
G	1	58.02879	6396.13539	64
M	2	189.06927	6339.11393	63
R	3	345.17038	6208.07344	62
K	4	473.26534	6051.97233	61
I	5	586.34941	5923.87737	60
V	6	685.41782	5810.79331	59
A	7	756.45493	5711.72489	58
G	8	813.4764	5640.68778	57
K	9	941.57136	5583.66631	56
L	10	1054.65542	5455.57135	55
Q	11	1182.714	5342.48729	54
T	12	1283.76168	5214.42871	53
G	13	1340.78314	5113.38103	52
A	14	1411.82026	5056.35957	51
D	15	1526.8472	4985.32246	50
F	16	1673.91561	4870.29551	49
E	17	1802.95821	4723.2271	48
G	18	1859.97967	4594.18451	47
S	19	1947.0117	4537.16304	46
K	20	2075.10666	4450.13101	45
G	21	2132.12812	4322.03605	44
G	22	2189.14959	4265.01459	43
C	23	2292.15877	4207.99312	42
K	24	2420.25374	4104.98394	41
A	25	2491.29085	3976.88898	40
S	26	2578.32288	3905.85186	39
G	27	2635.34434	3818.81983	38
G	28	2692.3658	3761.79837	37
A	29	2763.40292	3704.77691	36
V	30	2862.47133	3633.73979	35
V	31	2961.53975	3534.67138	34
E	32	3090.58234	3435.60297	33
N	33	3204.62527	3306.56037	32
S	34	3291.65729	3192.51745	31
H	35	3428.71621	3105.48542	30
N	36	3542.75913	2968.42651	29
A	37	3613.79625	2854.38358	28
G	38	3670.81771	2783.34647	27
P	39	3767.87047	2726.325	26
A	40	3838.90759	2629.27224	25
Y	41	4001.97092	2558.23513	24
C	42	4104.9801	2395.1718	23
V	43	4204.04851	2292.16261	22
G	44	4261.06998	2193.0942	21
Y	45	4424.13331	2136.07273	20
C	46	4527.14249	1973.00941	19
G	47	4584.16395	1870.00022	18
N	48	4698.20688	1812.97876	17
N	49	4812.24981	1698.93583	16
G	50	4869.27127	1584.8929	15
V	51	4968.33969	1527.87144	14
V	52	5067.4081	1428.80303	13
T	53	5168.45578	1329.73461	12
R	54	5324.55689	1228.68693	11
N	55	5438.59982	1072.58582	10
A	56	5509.63693	958.5429	9
N	57	5623.67986	887.50578	8
A	58	5694.71697	773.46286	7
N	59	5808.7599	702.42574	6
L	60	5921.84396	588.38281	5
A	61	5992.88107	475.29875	4
R	62	6148.98219	404.26164	3
T	63	6250.02986	248.16053	2
K	64	6378.12483	147.11285	1

Table S4 - Theoretical Mass fragments of peptides C2$_{A24}$ and C1$_{A22}$

C2$_{A24}$

		b	*y*	
G	1	58.02879	6396.13539	64
M	2	189.06927	6339.11393	63
R	3	345.17038	6208.07344	62
K	4	473.26534	6051.97233	61
I	5	586.34941	5923.87737	60
V	6	685.41782	5810.79331	59
A	7	756.45493	5711.72489	58
G	8	813.4764	5640.68778	57
K	9	941.57136	5583.66631	56
L	10	1054.65542	5455.57135	55
Q	11	1182.714	5342.48729	54
T	12	1283.76168	5214.42871	53
G	13	1340.78314	5113.38103	52
A	14	1411.82026	5056.35957	51
D	15	1526.8472	4985.32246	50
F	16	1673.91561	4870.29551	49
E	17	1802.95821	4723.2271	48
G	18	1859.97967	4594.18451	47
S	19	1947.0117	4537.16304	46
K	20	2075.10666	4450.13101	45
G	21	2132.12812	4322.03605	44
G	22	2189.14959	4265.01459	43
C	23	2292.15877	4207.99312	42
K	24	2420.25374	4104.98394	41
A	25	2491.29085	3976.88898	40
S	26	2578.32288	3905.85186	39
G	27	2635.34434	3818.81983	38
G	28	2692.3658	3761.79837	37
A	29	2763.40292	3704.77691	36
V	30	2862.47133	3633.73979	35
V	31	2961.53975	3534.67138	34
E	32	3090.58234	3435.60297	33
N	33	3204.62527	3306.56037	32
S	34	3291.65729	3192.51745	31
H	35	3428.71621	3105.48542	30
N	36	3542.75913	2968.42651	29
A	37	3613.79625	2854.38358	28
G	38	3670.81771	2783.34647	27
P	39	3767.87047	2726.325	26
A	40	3838.90759	2629.27224	25
Y	41	4001.97092	2558.23513	24
C	42	4104.9801	2395.1718	23
V	43	4204.04851	2292.16261	22
G	44	4261.06998	2193.0942	21
Y	45	4424.13331	2136.07273	20
C	46	4527.14249	1973.00941	19
G	47	4584.16395	1870.00022	18
N	48	4698.20688	1812.97876	17
N	49	4812.24981	1698.93583	16
G	50	4869.27127	1584.8929	15
V	51	4968.33969	1527.87144	14
V	52	5067.4081	1428.80303	13
T	53	5168.45578	1329.73461	12
R	54	5324.55689	1228.68693	11
N	55	5438.59982	1072.58582	10
A	56	5509.63693	958.5429	9
N	57	5623.67986	887.50578	8
A	58	5694.71697	773.46286	7
N	59	5808.7599	702.42574	6
L	60	5921.84396	588.38281	5
A	61	5992.88107	475.29875	4
R	62	6148.98219	404.26164	3
T	63	6250.02986	248.16053	2
K	64	6378.12483	147.11285	1

C1$_{A22}$

		b	*y*	
G	1	58.02879	6371.07139	64
M	2	189.06927	6314.04993	63
R	3	345.17038	6183.00944	62
K	4	473.26534	6026.90833	61
I	5	586.34941	5898.81337	60
V	6	685.41782	5785.72931	59
A	7	756.45493	5686.66089	58
G	8	813.4764	5615.62378	57
K	9	941.57136	5558.60232	56
L	10	1054.65542	5430.50735	55
Q	11	1182.714	5317.42329	54
T	12	1283.76168	5189.36471	53
G	13	1340.78314	5088.31704	52
A	14	1411.82026	5031.29557	51
D	15	1526.8472	4960.25846	50
F	16	1673.91561	4845.23152	49
E	17	1802.95821	4698.1631	48
G	18	1859.97967	4569.12051	47
S	19	1947.0117	4512.09905	46
K	20	2075.10666	4425.06702	45
W	21	2261.18597	4296.97205	44
G	22	2318.20744	4110.89274	43
A	23	2389.24455	4053.87128	42
V	24	2488.31296	3982.83417	41
C	25	2591.32215	3883.76575	40
S	26	2678.35418	3780.75657	39
G	27	2735.37564	3693.72454	38
S	28	2822.40767	3636.70307	37
T	29	2923.45535	3549.67105	36
A	30	2994.49246	3448.62337	35
V	31	3093.56087	3377.58625	34
A	32	3164.59799	3278.51784	33
N	33	3278.64092	3207.48073	32
S	34	3365.67294	3093.4378	31
H	35	3502.73186	3006.40577	30
N	36	3616.77478	2869.34686	29
A	37	3687.8119	2755.30393	28
G	38	3744.83336	2684.26682	27
P	39	3841.88612	2627.24536	26
A	40	3912.92324	2530.19259	25
Y	41	4075.98657	2459.15548	24
C	42	4178.99575	2296.09215	23
V	43	4278.06416	2193.08296	22
G	44	4335.08563	2094.01455	21
Y	45	4498.14896	2036.99309	20
C	46	4601.15814	1873.92976	19
G	47	4658.1796	1770.92057	18
N	48	4772.22253	1713.89911	17
N	49	4886.26546	1599.85618	16
G	50	4943.28692	1485.81326	15
V	51	5042.35534	1428.79179	14
V	52	5141.42375	1329.72338	13
T	53	5242.47143	1230.65497	12
R	54	5398.57254	1129.60729	11
N	55	5512.61547	973.50618	10
A	56	5583.65258	859.46325	9
N	57	5697.69551	788.42614	8
A	58	5768.73262	674.38321	7
N	59	5882.77555	603.34609	6
V	60	5981.84396	489.30317	5
A	61	6052.88107	390.23475	4
K	62	6180.97604	319.19764	3
T	63	6282.02372	191.10268	2
A	64	6353.06083	90.055	1

Table S5 - Theoretical Mass fragments of peptides C2$_{28-63}$ $[M+H]^+$ and C2$_{28-63}$ A41 $[M+H]^+$

C2$_{28-63}$

		b	*y*	
A	1	72.04444	3704.77691	36
V	2	171.11285	3633.73979	35
V	3	270.18126	3534.67138	34
E	4	399.22386	3435.60297	33
N	5	513.26678	3306.56037	32
S	6	600.29881	3192.51745	31
H	7	737.35772	3105.48542	30
N	8	851.40065	2968.42651	29
A	9	922.43776	2854.38358	28
G	10	979.45923	2783.34647	27
P	11	1076.51199	2726.325	26
A	12	1147.5491	2629.27224	25
Y	13	1310.61243	2558.23513	24
C	14	1413.62162	2395.1718	23
V	15	1512.69003	2292.16261	22
G	16	1569.71149	2193.0942	21
Y	17	1732.77482	2136.07273	20
C	18	1835.78401	1973.00941	19
G	19	1892.80547	1870.00022	18
N	20	2006.8484	1812.97876	17
N	21	2120.89133	1698.93583	16
G	22	2177.91279	1584.8929	15
V	23	2276.9812	1527.87144	14
V	24	2376.04962	1428.80303	13
T	25	2477.0973	1329.73461	12
R	26	2633.19841	1228.68693	11
N	27	2747.24133	1072.58582	10
A	28	2818.27845	958.5429	9
N	29	2932.32137	887.50578	8
A	30	3003.35849	773.46286	7
N	31	3117.40141	702.42574	6
L	32	3230.48548	588.38281	5
A	33	3301.52259	475.29875	4
R	34	3457.6237	404.26164	3
T	35	3558.67138	248.16053	2
K	36	3686.76634	147.11285	1

C2$_{28-63}$ A41

		b	*y*	
A	1	72.04444	3672.80484	36
V	2	171.11285	3601.76772	35
V	3	270.18126	3502.69931	34
E	4	399.22386	3403.6309	33
N	5	513.26678	3274.5883	32
S	6	600.29881	3160.54538	31
H	7	737.35772	3073.51335	30
N	8	851.40065	2936.45444	29
A	9	922.43776	2822.41151	28
G	10	979.45923	2751.37439	27
P	11	1076.51199	2694.35293	26
A	12	1147.5491	2597.30017	25
Y	13	1310.61243	2526.26305	24
A	14	1381.64955	2363.19973	23
V	15	1480.71796	2292.16261	22
G	16	1537.73942	2193.0942	21
Y	17	1700.80275	2136.07273	20
C	18	1803.81194	1973.00941	19
G	19	1860.8334	1870.00022	18
N	20	1974.87633	1812.97876	17
N	21	2088.91925	1698.93583	16
G	22	2145.94072	1584.8929	15
V	23	2245.00913	1527.87144	14
V	24	2344.07755	1428.80303	13
T	25	2445.12522	1329.73461	12
R	26	2601.22633	1228.68693	11
N	27	2715.26926	1072.58582	10
A	28	2786.30637	958.5429	9
N	29	2900.3493	887.50578	8
A	30	2971.38642	773.46286	7
N	31	3085.42934	702.42574	6
L	32	3198.51341	588.38281	5
A	33	3269.55052	475.29875	4
R	34	3425.65163	404.26164	3
T	35	3526.69931	248.16053	2
K	36	3654.79427	147.11285	1
